# Author Correction: Straightforward preparation of supramolecular Janus nanorods by hydrogen bonding of end-functionalized polymers

**DOI:** 10.1038/s41467-020-19082-4

**Published:** 2020-10-08

**Authors:** Shuaiyuan Han, Sandrine Pensec, Dijwar Yilmaz, Cédric Lorthioir, Jacques Jestin, Jean-Michel Guigner, Frédérick Niepceron, Jutta Rieger, François Stoffelbach, Erwan Nicol, Olivier Colombani, Laurent Bouteiller

**Affiliations:** 1 Sorbonne Université, CNRS, Institut Parisien de Chimie Moléculaire, UMR 8232, Equipe Chimie des Polymères, 75252 Paris, France; 2 Sorbonne Université, CNRS, Laboratoire de Chimie de la Matière Condensée de Paris, UMR 7574, 75252 Paris, France; 3 Laboratoire Léon Brillouin, UMR12 CEA-CNRS, Bât. 563, CEA Saclay, 91191 Gif-Sur-Yvette, France; 4 Sorbonne Université, CNRS, Institut de Minéralogie, de Physique des Matériaux et de Cosmochimie, UMR 7590-IRD-MNHN, 75252 Paris, France; 5 Institut des Molécules et Matériaux du Mans (IMMM), UMR 6283 CNRS Le Mans Université, Avenue Olivier Messiaen, 72085 Le Mans Cedex 9, France

**Keywords:** Supramolecular polymers, Nanoparticles

Correction to: *Nature Communications* 10.1038/s41467-020-18587-2, published online 21 September 2020.

The original version of this Article contained an error in Fig. 2d, in which the *x*-axis was wrongly labelled n_τ_ instead of using the correct label nτ. This has been corrected in both the PDF and HTML versions of the Article.

