# Supplementary material for: Straightforward preparation of supramolecular Janus nanorods by hydrogen bonding of end-functionalized polymers
Source: Nat Commun. 2020 Sep 21;11:4760. doi: 10.1038/s41467-020-18587-2 (PMC7506555; doi:10.1038/s41467-020-18587-2)
Supplement: Supplementary file 1 — Supplementary Information [file 41467_2020_18587_MOESM1_ESM.pdf]

SUPPLEMENTARY INFORMATION

for

# Straightforward preparation of supramolecular Janus nanorods by hydrogen bonding of end-functionalized polymers

Han et al.

Contents:

1. SUPPLEMENTARY METHODS 2

   1.1. MATERIALS AND ANALYSIS 2

   1.2. SYNTHESIS OF RAFT AGENTS 3

   1.3. POLYMERIZATIONS 11

   1.4. JANUS NANORODS PREPARATION 23

2. SUPPLEMENTARY NOTES 24

   2.1. JANUS NANORODS CHARACTERIZATIONS 24

   2.2. PATHWAY DEPENDENCY AND STABILITY OF JANUS NANORODS 34

   2.3. CHARACTERIZATION OF THE EMULSIONS 39

3. SUPPLEMENTARY REFERENCES 43

# 1. SUPPLEMENTARY METHODS

## 1.1. MATERIALS AND ANALYSIS

**Materials.** The following reagents were used without purification: 2-Ethylhexyl isocyanate (98%, Aldrich), 1,8-diisocyanatooctane (98%, Aldrich), 1,4-diisocyanatobutane (97%, Aldrich), 1,8-diaminooctane (98%, Aldrich), 1,4-diaminobutane (99%, Aldrich), methyl trioctylammonium chloride (MTOAC, Aliquat 336, Aldrich), carbon disulfide (99.9%, Aldrich), 2-methyl-1-propanethiol (92%, Aldrich), 6-amino-1-hexanol (94%, Acros), di-*tert*-butyl dicarbonate (99%, Acros), triethylamine (TEA, ≥ 99%, Sigma-Aldrich), acryloyl chloride (≥ 97%, Sigma-Aldrich), dimethylamine-$d_6$ hydrochloride (>98%, 99.3% atom D, Eurisotop), oxalyl chloride (98%, Alfa Aesar), HCl in dioxane (4M, Sigma-Aldrich), chloroform (≥99.9%, Carlo Erba), acetone (≥99.8%, Carlo Erba), dimethyl sulfoxide (DMSO, ≥99.9%, SeccoSolv).

Prior to use, 2,2'-azoisobutyronitrile (AIBN, 98%, Sigma-Aldrich) was recrystallized from methanol. *N,N*-Dimethylacrylamide (DMAc, 99%, Aldrich) and 4-acryloylmorpholine (NAM, 97%, Aldrich) were purified over basic alumina to remove the inhibitor. The anhydrous solvents [dichloromethane (DCM), diethyl ether (DE)] were obtained from a solvent purification system (MBraun SPS).

**NMR analysis.** $^{1}H$ and $^{13}C$ spectra were recorded on a Bruker Avance 400 spectrometer. DMSO-$D_6$ (99.80% D, Eurisotop) or $CDCl_3$ (99.50% D, Eurisotop) were used as solvents. Calibration was done using the chemical shift of the solvent residual resonance.

**Size exclusion chromatography (SEC).** Measurements were done by injecting 10 g $L^{-1}$ polymer solutions using dimethylformamide (DMF) containing 1 g $L^{-1}$ of LiBr as eluent. The flow rate was set at 0.8 mL $min^{-1}$ using a Waters HPLC 515 pump. Two columns thermostated at 60 °C (PSS GRAM, 1000 Å, 8 mm×300 mm and PSS GRAM, 30 Å, 8 mm×300 mm) were used for separation. Polymers were detected with a differential refractive index detector. Molar masses were computed with Omnisec v4.7 software, based on a PMMA calibration curve.

## 1.2. SYNTHESIS OF RAFT AGENTS

### 1.2.1. Synthesis of $NH_3Cl$-CTA

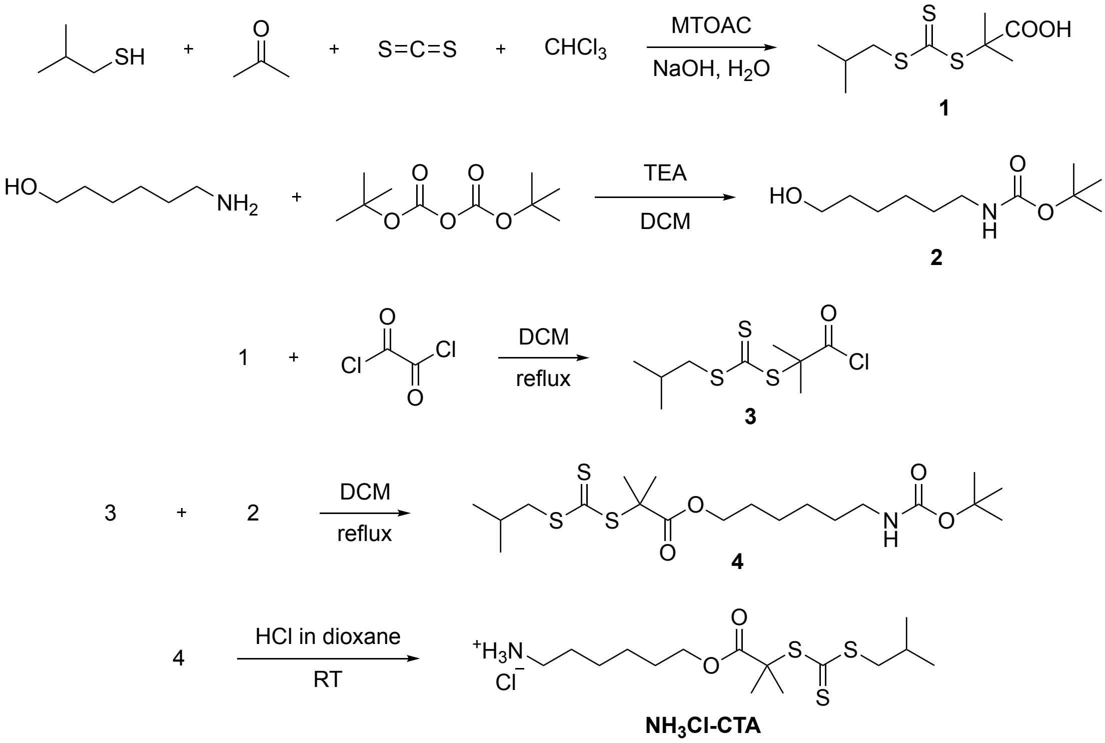


**Supplementary Figure 1:** Synthesis of **$NH_3Cl$-CTA**.

**Trithiocarbonate 1** was synthesized according to a procedure described by Winnik *et al.*[1] Under argon atmosphere and mechanical agitation, a NaOH aqueous solution (20 mL, 50 wt%, 250 mmol) was added dropwise to a solution of 2-methyl-1-propanethiol (24 mL, 221 mmol), acetone (140 mL, 1.9 mol) and methyltrioctylammonium chloride (4 mL, 22.5 mmol) kept in an ice-water bath. After the end of the addition, the reaction was stirred for 20 min. A solution of carbon disulfide (14 mL, 220 mmol) in acetone (40 mL) was slowly added into the mixture (within about 5 minutes), the solution was stirred for another 30 min. Chloroform (28 mL, 333 mmol) was added in one portion, and 65 mL of an aqueous NaOH solution (50 wt%, 815 mmol) was added dropwise. The reaction mixture was stirred overnight at room temperature. 325 mL of water was added to the resulting reaction mixture, followed by 165 mL of concentrated HCl to acidify the reaction to pH=1-2. The remaining acetone was removed by purging with argon. The yellow solid was collected by filtration and then washed with distilled water to remove the salt. The bright yellow solid (35.43 g, 58%) was obtained by recrystallization from an acetone/pentane (1/10) solution.

$^1$H NMR (400 MHz, $CDCl_3$) $\delta$ 11.25 (s br, -COO**H**, 1H), 3.21 (d, $J$ = 6.8 Hz, -C**H**$_2$S-, 2H), 2.04-1.92 (m, $(CH_3)_2$C**H**$CH_2$-, 1H), 1.72 (s, (C**H**$_3$)$_2$CS-, 6H), 1.01 (d, $J$ = 6.8Hz, (C**H**$_3$)$_2$CH$CH_2$-, 6H).

$^{13}$C NMR (100 MHz, $CDCl_3$) $\delta$ 221.09, 178.95, 55.76, 45.54, 28.01, 25.37, 22.22.

**Protected amino alcohol 2.** 6-Amino-1-hexanol (25 g, 213 mmol, 1 eq) was dissolved in 530 mL of anhydrous DCM in a round-bottom flask under argon. The solution was cooled to 0 °C in an ice bath. TEA (31.2 mL, 224 mmol, 1.05 eq) was added by syringe, then di-*tert*-butyl dicarbonate (51.4 mL, 224 mmol, 1.05 eq) was added slowly. The solution was allowed to reach room temperature and stirred overnight. DCM was evaporated under reduced pressure and the product was dissolved in DE. The solution was washed with 380 mL of 1M HCl and three times with 380 mL of distilled water. The organic layer was collected and dried with $MgSO_4$. 39.60 g (86%) of a white waxy solid was obtained.

$^1$H NMR (400 MHz, $CDCl_3$) $\delta$ 1.41-1.30 (m, $HOCH_2CH_2C\mathbf{H_2}C\mathbf{H_2}CH_2$-, 4H), 1.45 (s, $(C\mathbf{H_3})_3C$-, 9H), 1.52-1.46 (m, $HOCH_2C\mathbf{H_2}CH_2$-, 2H), 1.61-1.53 (m, -$HNCH_2C\mathbf{H_2}CH_2$-, 2H), 1.88 (s br, $\mathbf{H}OCH_2$-, 1H), 3.11 (t, $J$ = 7.2 Hz, -$HNC\mathbf{H_2}CH_2$-, 2H), 3.63 (t, $J$ = 7.2 Hz, $HOC\mathbf{H_2}CH_2$-, 2H), 4.55 (s br, -$CH_2N\mathbf{H}C$=O, 1H).

$^{13}$C NMR (100 MHz, $CDCl_3$) $\delta$ 156.21, 79.21, 62.78, 40.51, 32.72, 30.19, 28.55, 26.52, 25.42.

**Trithiocarbonate 4.** Adapted from Winnik *et al.*[1] Oxalyl chloride (18 mL, 150 mmol, 3 eq) was added dropwise to a solution of **1** (12.7 g, 50 mmol, 1eq) in 50 mL of dry DCM under argon at room temperature. The mixture was refluxed for 1-2 hours until a dark reddish solution was formed. The excess oxalyl chloride and DCM were evaporated under vacuum to obtain **3**. A solution of **2** (16.3 g, 75 mmol, 1.5 eq) in 50 mL of dry DCM was added dropwise on **3** contained in a flask equipped with a reflux condenser. The solution was refluxed overnight, cooled to room temperature and DCM was removed in vacuo. The remaining oil was dissolved into 500 mL of DE, washed successively with saturated $NaHCO_3$, brine, water and dried over anhydrous $MgSO_4$. After removing the solvent, the crude product was purified over a silica gel column using ethyl acetate / petroleum ether = 1/4 as eluent. 11.46 g (51%) of a yellow viscous liquid was obtained.

$^1$H NMR (400 MHz, $CDCl_3$) $\delta$ 4.50 (s br, -$CH_2N\mathbf{H}COO$-, 1H), 4.07 (t, $J$ = 6.6 Hz, -$OC\mathbf{H_2}CH_2$-, 2H), 3.18 (d, $J$ = 6.8 Hz, -$CHC\mathbf{H_2}S$-, 2H), 3.09 (m, $J$ = 6.6 Hz, -$CH_2C\mathbf{H_2}NH$-, 2H), 2.04-1.87 (m, $(CH_3)_2C\mathbf{H}CH_2$-, 1H), 1.68 (s, $(C\mathbf{H_3})_2CS$-, 6H), 1.65-1.55 (m, -$OCH_2C\mathbf{H_2}CH_2$-, 2H), 1.46 (m, -$NHCH_2C\mathbf{H_2}CH_2$-, 2H), 1.43 (s, $(C\mathbf{H_3})_3C$-, 9H), 1.37-1.27 (m, -$CH_2C\mathbf{H_2}C\mathbf{H_2}CH_2$-, 4H), 0.99 (d, $J$ = 6.6 Hz, $(C\mathbf{H_3})_2CH$-, 6H).

$^{13}$C NMR (100 MHz, $CDCl_3$) $\delta$ 221.73, 173.09, 156.08, 79.16, 66.06, 56.17, 45.32, 40.63, 30.11, 28.57, 28.42, 28.07, 26.52, 25.78, 25.52, 22.16.

**$NH_3Cl$-CTA. 4** (11.30 g, 25 mmol, 1eq) was placed in a round-bottom flask in an ice bath under argon. 19 mL of 4 M HCl in dioxane was added slowly. The mixture was stirred at room temperature overnight. Dioxane was evaporated under reduced pressure, affording a bright yellow oil (9.56 g, 99%).

$^1$H NMR (400 MHz, $CDCl_3$) $\delta$ 8.30 (s br, -$CH_2N\mathbf{H_3}Cl$, 3H), 4.07 (t, $J$ = 6.6 Hz, -$OC\mathbf{H_2}CH_2$-, 2H), 3.20 (d, $J$ = 6.8 Hz, -$CHC\mathbf{H_2}S$-, 2H), 3.08-3.92 (m, -$CH_2C\mathbf{H_2}NH_3Cl$, 2H), 2.02-1.90 (m, $(CH_3)_2C\mathbf{H}CH_2$-, 1H), 1.83-1.72 (m, -$OCH_2C\mathbf{H_2}CH_2$-, 2H), 1.68 (s, $(C\mathbf{H_3})_2CS$-, 6H), 1.67-1.59 (m, $ClNH_3CH_2C\mathbf{H_2}CH_2$-, 2H), 1.50-1.32 (m, -

$CH_2C\mathbf{H_2}C\mathbf{H_2}CH_2$-, 4H), 1.00 (d, $J$ = 6.6 Hz, (C$\mathbf{H_3}$)$_2$CH-, 6H).

$^{13}$C NMR (100 MHz, $CDCl_3$) $\delta$221.73, 173.11, 65.81, 56.16, 45.31, 39.96, 28.19, 28.05, 27.55, 26.17, 25.49, 25.39, 22.15.

### 1.2.2. Synthesis of chain transfer agent 4/8-CTA

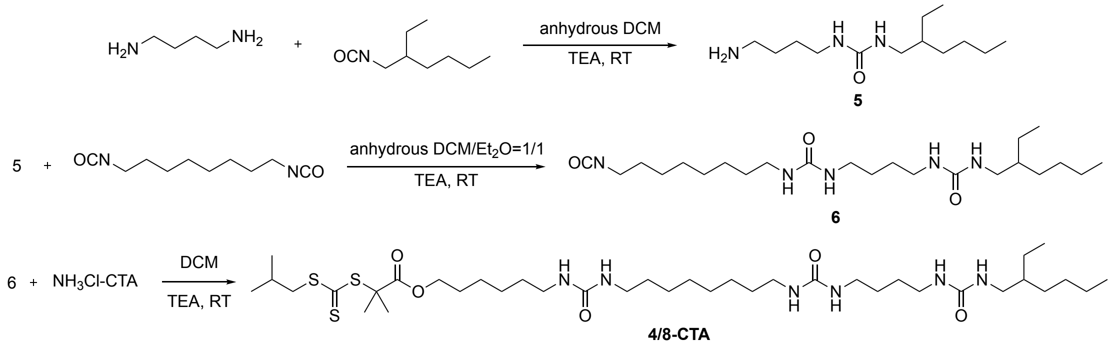


**Supplementary Figure 2:** Synthesis of **4/8-CTA**.

**1-(4-Aminobutyl)-3-(2-ethylhexyl)urea (5).** A solution of 2-ethylhexyl isocyanate (5 g, 32.2 mmol, 1eq) in dry DCM (25 mL) was added slowly (5 mL $h^{-1}$) under argon atmosphere into a round-bottom flask with a DCM (250 mL) solution of 1,4-diaminobutane (28.4 g, 322 mmol, 10 eq) and TEA (44 mL, 322 mmol, 10 eq). The solution was stirred overnight at room temperature. The reaction was completed when the -C$\mathbf{H_2}$NCO peak (3.3 ppm) on the NMR spectrum had disappeared. The solution was concentrated to about 75 mL. 150 mL of pentane was added and the mixture was centrifuged. The lower phase was recovered, the solvent evaporated and the crude product was purified over a silica gel column using MeOH/DCM = 1/7 as eluent. 6.42 g (82%) of a colorless viscous liquid was obtained.

$^1$H NMR (400 MHz, $CDCl_3$) $\delta$ 4.98 (s br, -N**H**$CH_2$CH-, 1H), 4.70 (t, $J$ = 5.8 Hz, -$CH_2CH_2$N**H**-, 2H), 3.23-3.13 (m, -NHC$\mathbf{H_2}$CH-, 2H), 3.13-3.00 (m, -NHC$\mathbf{H_2}CH_2$-, 2H), 2.80-2.69 (m, $NH_2$C$\mathbf{H_2}$-, 2H), 2.60 (s br, N$\mathbf{H_2}CH_2$-, 2H), 1.61-1.46 (m, $NH_2CH_2$C$\mathbf{H_2}$C$\mathbf{H_2}$-, 4H), 1.44-1.20 (m, -C**H**(C$\mathbf{H_2}CH_3$)(C$\mathbf{H_2}$C$\mathbf{H_2}$C$\mathbf{H_2}CH_3$), 9H), 0.85 (m, -CH($CH_2$C$\mathbf{H_3}$)($CH_2CH_2CH_2$C$\mathbf{H_3}$), 6H).

$^{13}$C NMR (100 MHz, $CDCl_3$) $\delta$158.92, 43.55, 41.52, 40.33, 39.95, 31.12, 29.98, 29.07, 27.78, 24.30, 23.19, 14.22, 11.03.

**1-(4-(3-(2-Ethylhexyl)ureido)butyl)-3-(8-isocyanatooctyl)urea (6).** A solution of **5** (6.25 g, 25.7 mmol, 1 eq) and TEA (7.14 mL, 51.4 mmol, 2 eq) in a dry mixture of solvents (70 mL, DCM/DE=1/1) was added to a stirred solution of 1,8-diisocyanatooctane (10.09 g, 51.4 mmol, 2 eq) in a dry mixture of solvents (250 mL, DCM/DE=1/1) under argon at room temperature. During the reaction a white precipitate appeared. The reaction was followed by $^1$H NMR to check the disappearance of the -C$\mathbf{H_2}NH_2$ peak at 2.80-2.69 ppm. At the end of the reaction, the reaction mixture was filtered, the white solid obtained was washed three times with the solvent mixture (3×100 mL, DCM/DE=1/1). The solid was dissolved into DCM and filtered. DCM was

evaporated from the filtrate. The resulting white solid was dried under vacuum overnight to obtain 3.05 g (27%).

$^{1}$H NMR (400 MHz, $CDCl_3$) $\delta$ 4.78-4.66 (m, OCN($CH_2$)$_8$N**H**CON**H**($CH_2$)$_4$NHCONH-, 2H), 4.55 (t, $J$ = 5.6 Hz, -CHC$H_2$N**H**-, 1H), 4.48 (t, $J$ = 5.6 Hz, NHCONH($CH_2$)$_4$N**H**CONHC$H_2$CH-, 1H), 3.31 (t, $J$ = 6.7 Hz, OCNC**H$_2$**-, 2H), 3.28-3.20 (m, OCN($CH_2$)$_7$C**H$_2$**NHCONHC**H$_2$**($CH_2$)$_3$-, 4H), 3.20-3.14 (m, NHC**H$_2$**CH-, 2H), 3.14-3.06 (m, NHCONH($CH_2$)$_3$C**H$_2$**NHCO-, 2H), 1.67-1.60 (m, OCNC$H_2$C**H$_2$**-, 2H), 1.57-1.46 (m, -NHC$H_2$(C**H$_2$**)$_2$C$H_2$NH-, -C$H_2$C**H**-, 5H), 1.45-1.26 (m, OCN($CH_2$)$_2$(C**H$_2$**)$_5$-, -CH(C**H$_2$**C$H_3$)((C**H$_2$**)$_3$C$H_3$), 18H), 0.96-0.86 (m, -CH(C$H_2$C**H$_3$**)(($CH_2$)$_3$C**H$_3$**), 6H).

$^{13}$C NMR (100 MHz, $CDCl_3$) $\delta$ 159.68, 159.50, 122.10, 43.43, 43.10, 40.45, 40.20, 40.08, 31.40, 31.12, 30.60, 29.43, 29.11, 29.07, 28.23, 28.12, 27.07, 26.64, 24.26, 23.22, 14.23, 11.01.

**4/8-CTA. 6** (2.83g, 6.43 mmol, 1 eq), **NH$_3$Cl-CTA** (3.63 g, 9.35 mmol, 1.5 eq) and 900 mL of anhydrous DCM were stirred in a dried 1L two-necked flask under argon. TEA (0.6955 g, 6.87 mmol, 1.1 eq) dissolved in 21 mL of anhydrous DCM was injected into the two-necked flask using a syringe at a flow rate of 1.5 mL $h^{-1}$. The reaction was stirred overnight at room temperature. The precipitate was filtered with a Büchner funnel and washed with DCM. A yellow powder (2.80 g, 55%) was obtained.

$^{1}$H NMR (400 MHz, DMSO-$d_6$, 60 °C) $\delta$ 5.75-5.65 (m, -N**H**, 6H), 4.00 (t, $J$ = 6.3 Hz, -OC**H$_2$**-, 2H), 3.23 (d, $J$ = 6.8Hz, -SC**H$_2$**CH-, 2H), 3.04-2.86 (m, -C**H**$_2$NH-, 12H), 1.96-1.84 (m, (-SC$H_2$C**H**(C$H_3$)$_2$, 1H), 1.63 (s, (C**H$_3$**)$_2$C-, 6H), 1.58-1.48 (m, C$H_3$C$H_2$C**H**-, 1H), 1.40-1.12 (m, -CH(C**H$_2$**C$H_3$)((C**H$_2$**)$_3$C$H_3$), -NHC$H_2$(C**H$_2$**)$_2$C$H_2$NH-, -NHC$H_2$(C**H$_2$**)$_6$C$H_2$NH-, -NHC$H_2$(C**H$_2$**)$_4$C$H_2$O-, 32H), 0.95 (d, $J$ = 6.8 Hz, (C**H$_3$**)$_2$CH-, 6H), 0.89-0.79 (m, -CH(C$H_2$C**H$_3$**)(($CH_2$)$_3$C**H$_3$**), 6H). See Supplementary Figure 3.

$^{13}$C NMR (100 MHz, DMSO-$d_6$, 80 °C) $\delta$ 221.46, 171.20, 157.94, 157.80, 65.06, 55.85, 44.30, 42.02, 30.25, 29.61, 29.55, 28.32, 28.10, 27.52, 27.21, 27.10, 25.94, 25.59, 24.77, 24.71, 23.49, 22.00, 21.09, 13.30, 10.32 (some peaks overlap with DMSO). See Supplementary Figure 4.

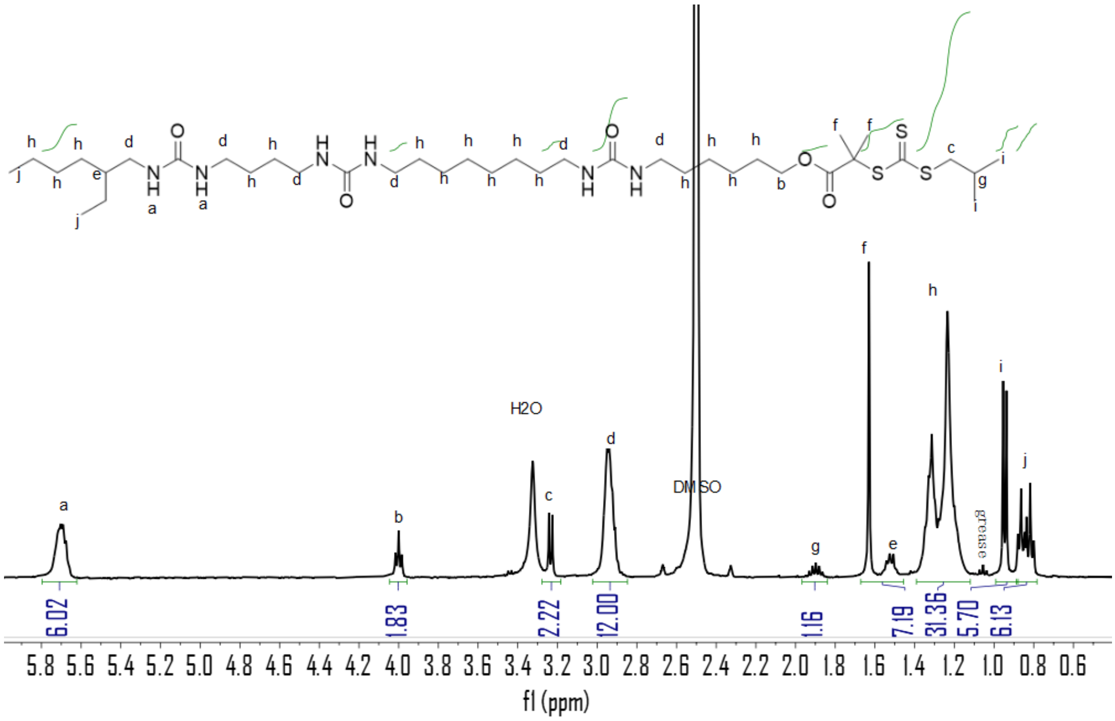

**Supplementary Figure 3:** $^{1}$H NMR spectrum of **4/8-CTA** (DMSO-d$_6$, 60 °C).

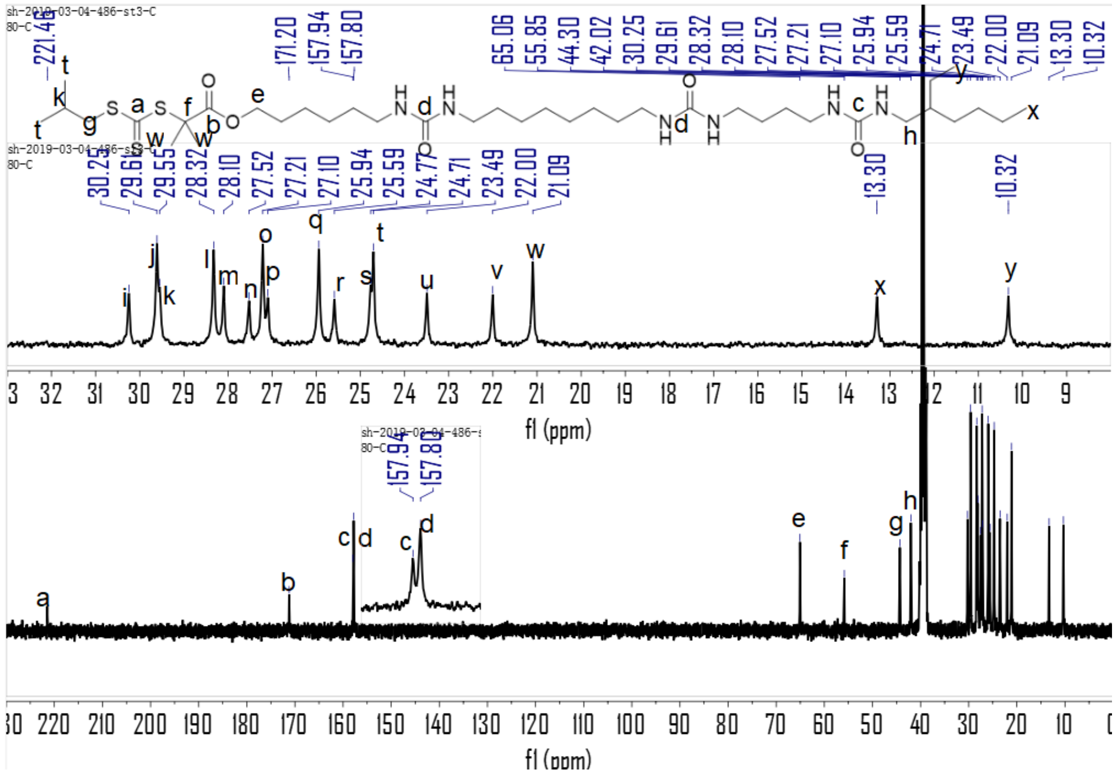

**Supplementary Figure 4:** $^{13}$C NMR spectrum of **4/8-CTA** (DMSO-d$_6$, 80 °C).

### 1.2.3. Synthesis of chain transfer agent 8/4-CTA

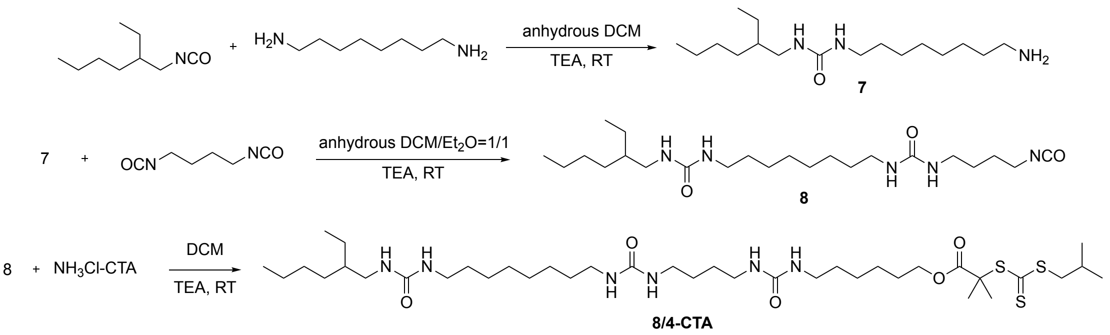


**Supplementary Figure 5:** Synthesis of **8/4-CTA**.

**1-(8-aminooctyl)-3-(2-ethylhexyl)urea (7).** A solution of 2-ethylhexyl isocyanate (5 g, 32.2 mmol, 1eq) in dry DCM (25 mL) was added slowly (5 mL $h^{-1}$) under argon atmosphere into a round-bottom flask containing a DCM (250 mL) solution of 1,8-diaminooctane (46.45 g, 322 mmol, 10 eq) and TEA (5 mL, 36.6 mmol, 1.13 eq). The solution was stirred overnight at room temperature. The reaction was completed when the -C**H**$_2$NCO peak (3.3 ppm) on the NMR spectrum had disappeared. The solution was concentrated to about 75 mL. 150 mL of pentane was added and the mixture was centrifuged. The lower phase was recovered, the solvent evaporated and the crude product was purified over a silica gel column using MeOH/DCM = 1/7 as eluent. 6.10 g (63%) of a colorless viscous liquid was obtained.

$^1$H NMR (400 MHz, CDCl$_3$) $\delta$ 4.66 (t, $J$ = 5.8 Hz, -N**H**CH$_2$CH-, 1H), 4.61 (t, $J$ = 5.8 Hz, -CH$_2$CH$_2$N**H**-, 2H), 3.18-3.09 (m, -NHC**H**$_2$CH-, 2H), 3.09-3.04 (m, -NHC**H**$_2$CH$_2$-, 2H), 2.67 (t, $J$ = 7.0 Hz, NH$_2$C**H**$_2$CH$_2$-, 2H), 1.87 (s br, N**H**$_2$CH$_2$-, 2H), 1.53-1.20 (m, NH$_2$CH$_2$(C**H**$_2$)$_6$-, -C**H**(C**H**$_2$CH$_3$)(C**H**$_2$C**H**$_2$C**H**$_2$CH$_3$), 21H), 0.92-0.82 (m, -CH(CH$_2$C**H**$_3$)(CH$_2$CH$_2$CH$_2$C**H**$_3$), 6H).

$^{13}$C NMR (100 MHz, CDCl$_3$) $\delta$ 158.72, 43.44, 42.02, 40.55, 39.81, 33.23, 31.00, 30.24, 29.30, 29.21, 28.94, 26.78, 26.72, 24.19, 23.04, 14.07, 10.90.

**1-(2-Ethylhexyl)-3-(8-(3-(4-isocyanatobutyl)ureido)octyl)urea (8).** A solution of **7** (5.82 g, 19.4 mmol, 1 eq) and TEA (5.4 mL, 38.9 mmol, 2 eq) in a dry mixture of solvents (64 mL, DCM/DE=1/1) was added to a stirred solution of 1,4-diisocyanatobutane (5.45 g, 38.9 mmol, 2 eq) in a dry mixture of solvents (220 mL, DCM/DE=1/1) under argon at room temperature. During the reaction a white precipitate appeared. The reaction was followed by $^1$H NMR to check the disappearance of the -C**H**$_2$NH$_2$ peak at 2.67 ppm. The reaction mixture was filtered, the white solid obtained was washed three times with the solvent mixture (3×90 mL, DCM/DE=1/1). The solid was dissolved in DCM and filtered. The filtrate was evaporated and the resulting white solid was dried under vacuum overnight to obtain 2.01 g (24%).

$^1$H NMR (400 MHz, CDCl$_3$) $\delta$ 5.41-4.69 (m, -CH$_2$N**H**-, 4H), 3.33 (t, $J$ = 6.7 Hz, OCNC**H**$_2$-, 2H), 3.25-3.05 (m, -C**H**$_2$NH-, 8H), 1.75-1.52 (m, OCNCH$_2$C**H**$_2$-, CH$_3$CH$_2$C**H**-, NHCH$_2$C**H**$_2$CH$_2$CH$_2$NCO, 5H), 1.51-1.38 (m, NHCH$_2$C**H**$_2$-, 4H), 1.38-1.22 (m, -(C**H**$_2$)$_3$CH$_3$, -CHC**H**$_2$CH$_3$ -NH(CH$_2$)$_2$(C**H**$_2$)$_4$, 16H), 0.94-0.83 (m, -CHCH$_2$C**H**$_3$, -(CH$_2$)$_3$C**H**$_3$), 6H).

$^{13}$C NMR (100 MHz, $CDCl_3$) $\delta$ 159.41, 159.28, 122.08, 43.40, 42.89, 40.33, 40.26, 40.05, 39.58, 31.14, 30.29, 30.24, 29.11, 29.00, 28.76, 27.78, 26.70, 26.61, 24.30, 23.20, 14.23, 11.05.

**8/4-CTA. 8** (1.73g, 3.93 mmol, 1 eq), **$NH_3Cl$-CTA** (2.22 g, 5.79 mmol, 1.5 eq) and 700 mL of anhydrous DCM were stirred in a dried 1 L two-necked flask under argon. TEA (0.42 g, 4.15 mmol, 1.1 eq) dissolved in 20 mL of anhydrous DCM were injected into the two-necked flask using a syringe with a flow rate of 1.5 mL $h^{-1}$. The reaction was stirred overnight at room temperature. The precipitate formed was filtered with a Büchner funnel and washed with DCM. A yellow powder (1.65 g, 53%) was obtained.

$^{1}$H NMR (400 MHz, DMSO-$d_6$, 60 °C) $\delta$ 5.77-5.61 (m, -N**H**, 6H), 4.00 (t, $J$ = 6.3 Hz, -OC**$H_2$**-, 2H), 3.23 (d, $J$ = 6.8Hz, -SC**$H_2$**CH-, 2H), 3.02-2.84 (m, -C**$H_2$**NH-, 12H), 1.96-1.84 (m, -$SCH_2$C**H**$(CH_3)_2$, 1H), 1.63 (s, (C**$H_3$**)$_2$C-, 6H), 1.54-1.47 (m, ($CH_3CH_2$C**H**-, 1H), 1.40-1.12 (m, -CH(C**$H_2$**CH3)((C**$H_2$**)$_3CH_3$), -$NHCH_2$(C**$H_2$**)$_6CH_2$NH-, -$NHCH_2$(C**$H_2$**)$_2CH_2$NH-, -$NHCH_2$(C**$H_2$**)$_4CH_2$O-, 32H), 0.95 (d, $J$ = 6.8 Hz, (C**$H_3$**)$_2$CH-, 6H), 0.89-0.79 (m, -CH($CH_2$C**$H_3$**)(($CH_2$)$_3$C**$H_3$**), 6H). See Supplementary Figure 6.

$^{13}$C NMR (100 MHz, DMSO-$d_6$, 60 °C) $\delta$ 221.65, 171.37, 158.01, 157.86, 65.18, 55.88, 44.31, 41.94, 30.28, 29.76, 29.68, 28.48, 28.20, 27.62, 27.33, 27.22, 26.05, 25.72, 24.90, 24.78, 23.54, 22.17, 21.25, 13.51, 10.49 (some peaks overlap with DMSO). See Supplementary Figure 7.

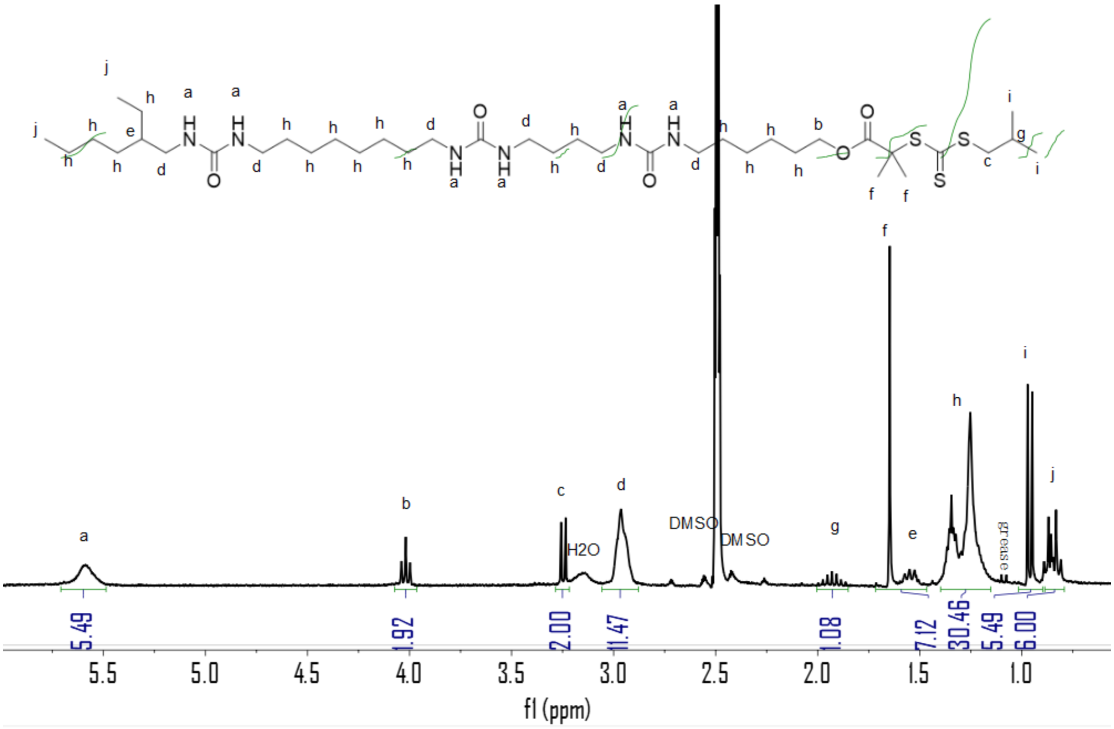


**Supplementary Figure 6:** $^{1}$H NMR spectrum of **8/4-CTA** (DMSO-$d_6$, 60 °C).

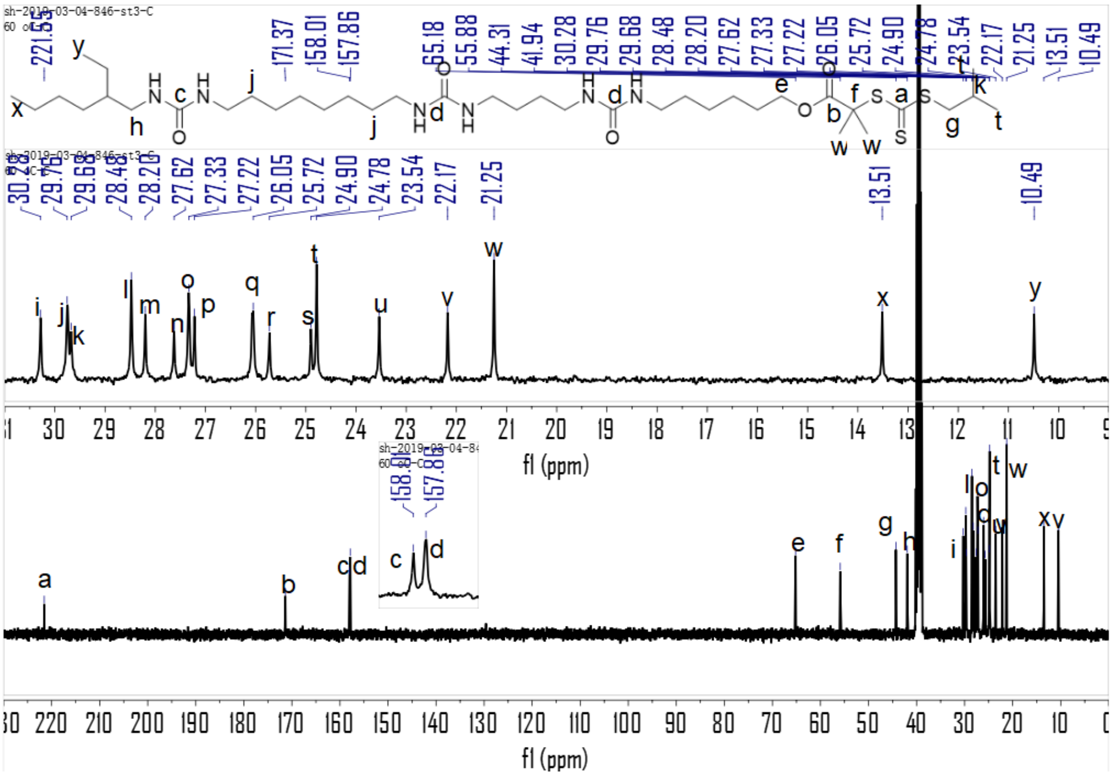

**Supplementary Figure 7:** $^{13}$C NMR spectrum of **8/4-CTA** (DMSO-$d_6$, 60 °C).

## 1.3. POLYMERIZATIONS

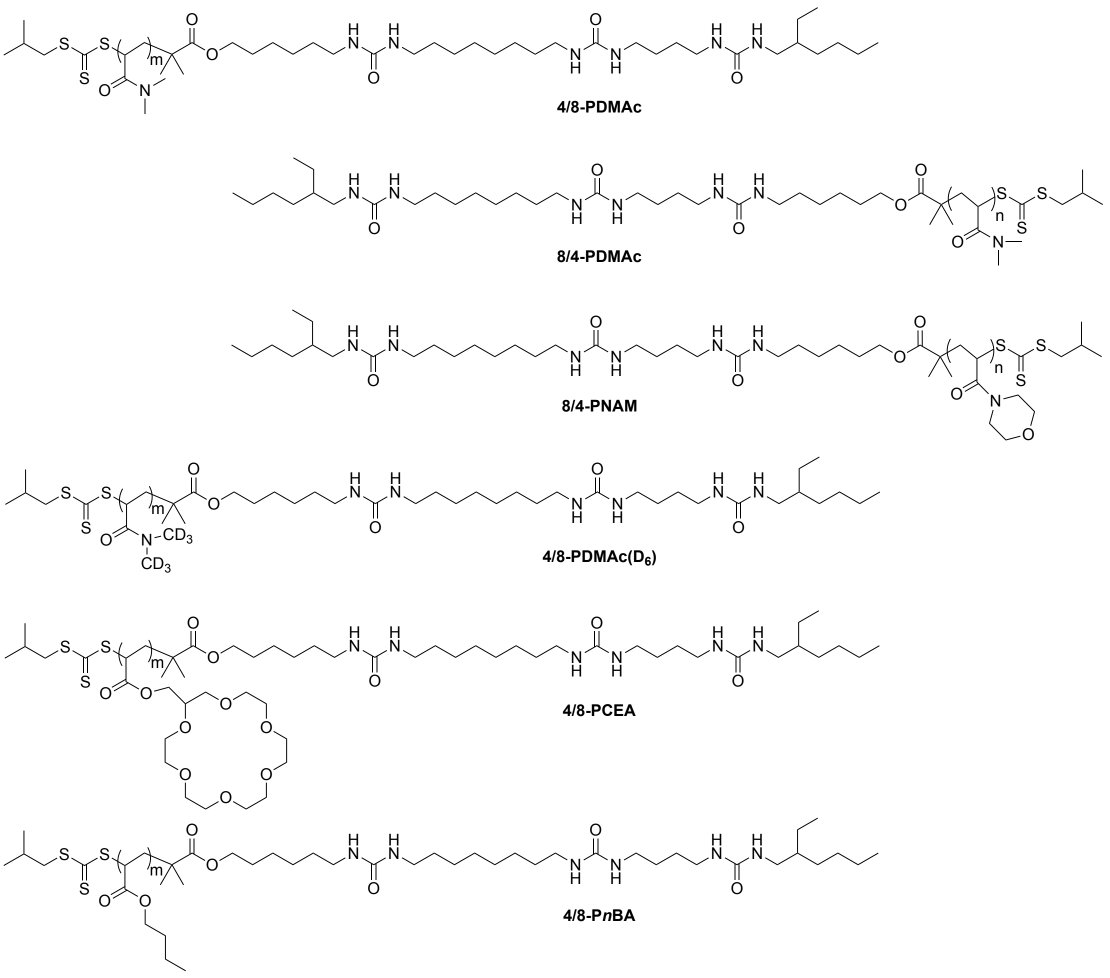


**Supplementary Figure 8:** Structure of the polymers.

**RAFT polymerization of *N,N*-dimethylacrylamide (DMAc)** based on **4/8-CTA** (or **8/4-CTA**). **4/8-CTA** (0.101 g, 0.128 mmol, 1eq), DMSO (7.5 mL) and DMAc (0.560 g, 5.65 mmol, 44 eq) were introduced in a Schlenk flask. In order to obtain a homogeneous solution, the mixture was heated to 75 °C and was degassed by bubbling argon for 30 min. AIBN (2.1 mg, 0.013 mmol, 0.1 eq) was introduced into the solution under argon, then the mixture was degassed for another 5 min. Samples were withdrawn periodically from the reaction mixture to determine the conversion by $^1$H NMR. After 72% conversion, the Schlenk was opened to air and cooled down to room temperature. The product was precipitated in about 100 mL of DE, filtered and was dissolved in water to perform dialysis with a 1 kDa membrane. A light-yellow powder (320 mg, 63%) was obtained by freeze-drying the dialyzed solution.

$^1$H NMR (400 MHz, DMSO-d$_6$) $\delta$ 5.79-5.63 (m, -N**H**, 6H), 4.00-3.85 (m, -OC**H$_2$**-, 2H), 3.13-2.68 (m, -C**H$_2$**NH-, -N(C**H$_3$**)$_2$, 204H), 2.65-2.12 (m, -CHC**H$_2$**S-, -C**H**(C=O)N(CH$_3$)$_2$), 1.96-1.84 (br, -C**H**(CH$_3$)$_2$, 1H), 1.75-1.04 (m, -C**H$_2$**CH(C=O)N(CH$_3$)$_2$, -CH$_2$C**H**(C**H$_2$**CH$_3$)(C**H$_2$**)$_3$CH$_3$, -OCH$_2$(C**H$_2$**)$_4$CH$_2$, -

NH(C=O)NH$CH_2$(C**H**$_2$)$_6$, -NH(C=O)NH$CH_2$(C**H**$_2$)$_2$-, -C(C**H**$_3$)$_2$, 96H), 1.02-0.93 (m, -CH(C**H**$_3$)$_2$, 6H), 0.92-0.77 (m, -$CH_2$C**H**$_3$, 6H). See Supplementary Figures 9 and 10.

SEC: see Supplementary Figure 21 and Supplementary Table 1.

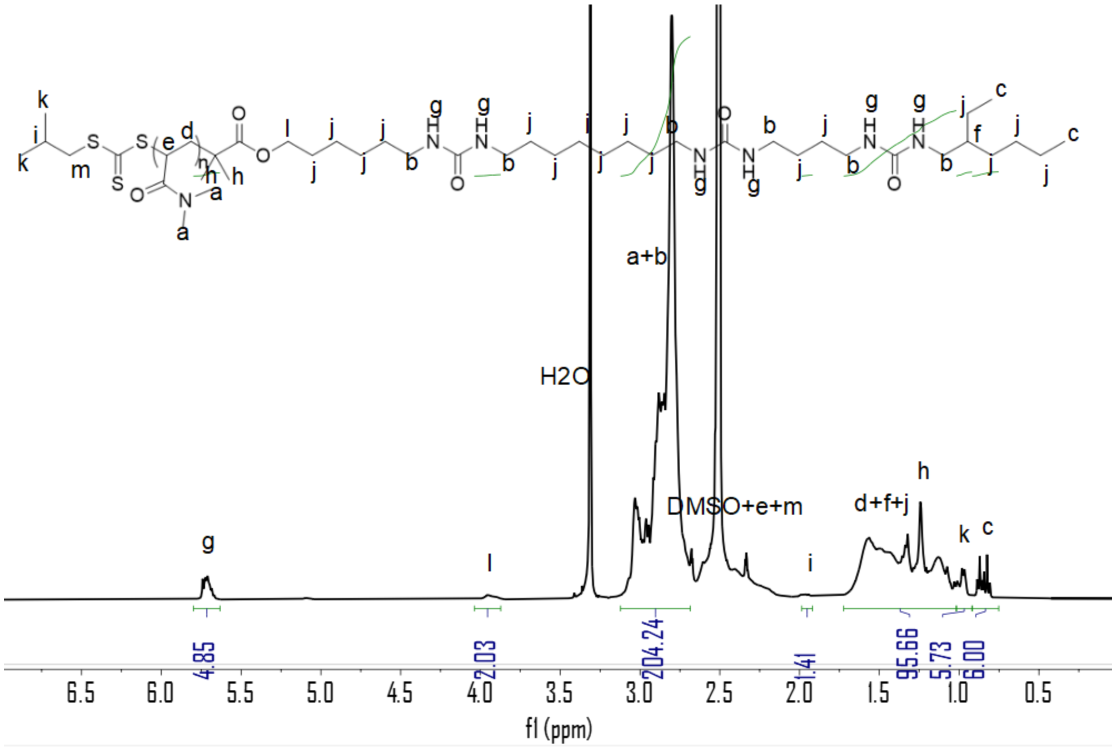


**Supplementary Figure 9:** $^1$H NMR spectrum of **4/8-PDMAc** (DMSO-d$_6$).

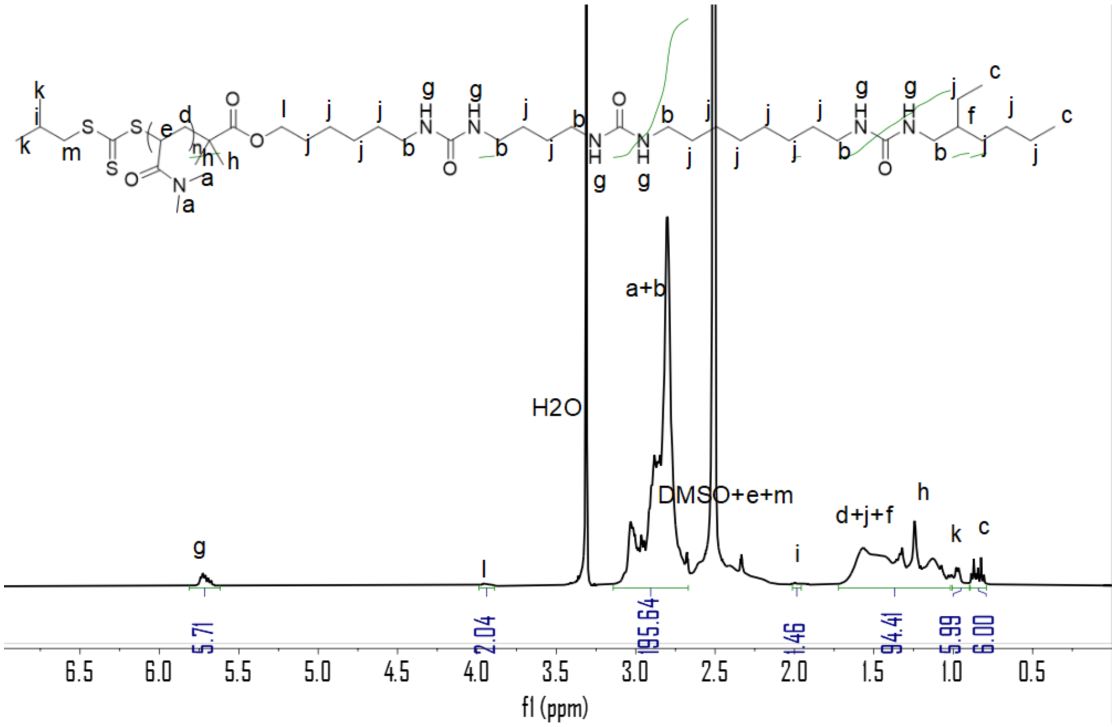


**Supplementary Figure 10:** $^1$H NMR spectrum of **8/4-PDMAc** (DMSO-$d_6$).

**RAFT polymerization of 4-acryloylmorpholine (NAM). 8/4-CTA** (0.101 g, 0.128 mmol, 1eq), DMSO (7.5 mL) and NAM (0.789 g, 559 mmol, 44 eq) were introduced in a Schlenk flask. In order to obtain a homogeneous solution, the mixture was heated to 75 °C and was degassed by bubbling argon for 30 min. AIBN (2.1 mg, 0.013 mmol, 0.1 eq) was introduced into the solution under argon, and the mixture was degassed for another 5 min. Samples were withdrawn periodically from the reaction mixture to determine conversion by $^1$H NMR. After 89% conversion, the Schlenk was opened to air and cooled down to room temperature. The product was precipitated in about 100 mL of DE and filtered. The powder was dissolved in water and filtered. The filtrate was dialyzed through a 1 kDa membrane. A light-yellow powder (480 mg, 68%) was obtained by freeze-drying the dialyzed solution.

$^1$H NMR (400 MHz, DMSO-$d_6$) $\delta$ 5.79-5.62 (m, -N**H**, 6H), 4.02-3.07 (m, -(C=O)OC**H₂**-, -NC**H**₂C**H**₂O-), 3.02-2.87 (m, -C**H**₂NH-, 12H), 2.65-2.12 (m, -CHC**H**₂S-, -C**H**(C=O)N(CH₃)₂), 1.96-1.02 (m, -C**H**₂CH(C=O)N(CH₃)₂, -C**H**(C**H**₂CH₃)(C**H**₂)₃CH₃, -C**H**(CH₃)₂, -OCH₂(C**H**₂)₄CH₂, -NH(C=O)NHCH₂(C**H**₂)₂-, -NH(C=O)NHCH₂(C**H**₂)₆-, -C(C**H**₃)₂, 132H), 1.01-0.94 (m, -CH(C**H**₃)₂, 6H), 0.92-0.77 (m, -CH₂C**H**₃, 6H). See Supplementary Figure 11.

SEC: see Supplementary Figure 21 and Supplementary Table 1.

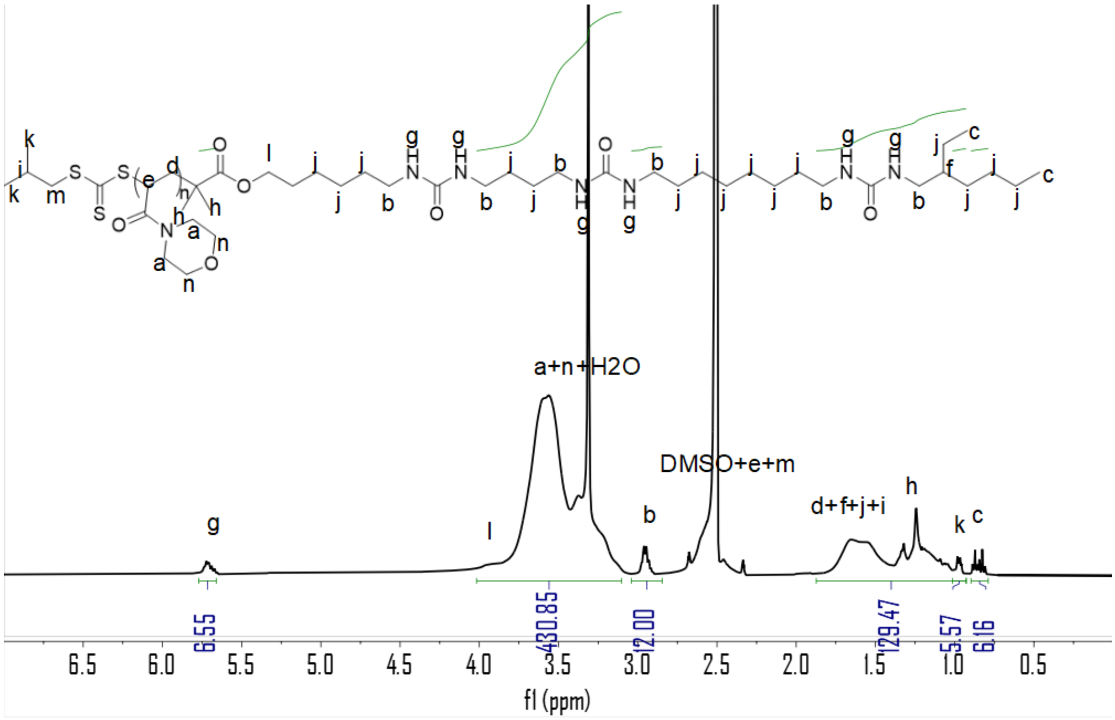


**Supplementary Figure 11:** $^{1}H$ NMR spectrum of **8/4-PNAM** (DMSO-$d_6$).

**Synthesis of 4/8-PDMAc($D_6$)**

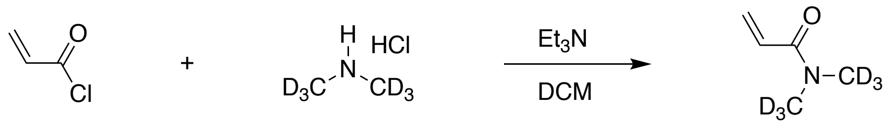


**Supplementary Figure 12:** Synthesis of **DMAc($D_6$)**.

**DMAc($D_6$).** Dimethylamine-$D_6$ hydrochloride (10 g, 114 mmol, 1 eq) was dissolved in 180 mL dried DCM under argon and cooled to 0 °C. TEA (34.9 mL, 250 mmol, 2.2 eq) was slowly added into the DCM solution (15 mL $h^{-1}$) under argon at 0 °C. Then acryloyl chloride (10 mL, 123 mmol, 1.1 eq) dissolved in 50 mL DCM was injected at 15 mL $h^{-1}$ under argon at 0 °C. After completion of the reaction, the flask was removed from the ice bath and stirred at room temperature for 36 h. The mixture was put in the freezer (-30 °C) and quickly filtered to remove the solid. The filtrate was concentrated and purified by column chromatography (acetone/DCM=1/9) to give a yellow product. A colorless liquid (6.5 g, 54%) was obtained after distillation with a Kugelrohr apparatus.

$^1$H NMR (400 MHz, $CDCl_3$) $\delta$ 6.54 (dd, $J$ = 16.8, 10.5 Hz, C**H**=$CH_2$, 1H), 6.25 (dd, $J$ = 16.8, 2.0 Hz, CH=C**H**(c)H, 1H), 5.62 (dd, $J$ = 10.5, 2.0 Hz, CH=CH**H**(t), 1H). See Supplementary Figure 13.

$^{13}$C NMR (100 MHz, $CDCl_3$) $\delta$ 163.37, 126.44, 124.14, 34.49-32.95, 32.65-31.19. See Supplementary Figure 14.

ESI-MS (m/z): 128.08 [M + Na]+, 128.10 calculated for $C_5H_3D_6NONa$.

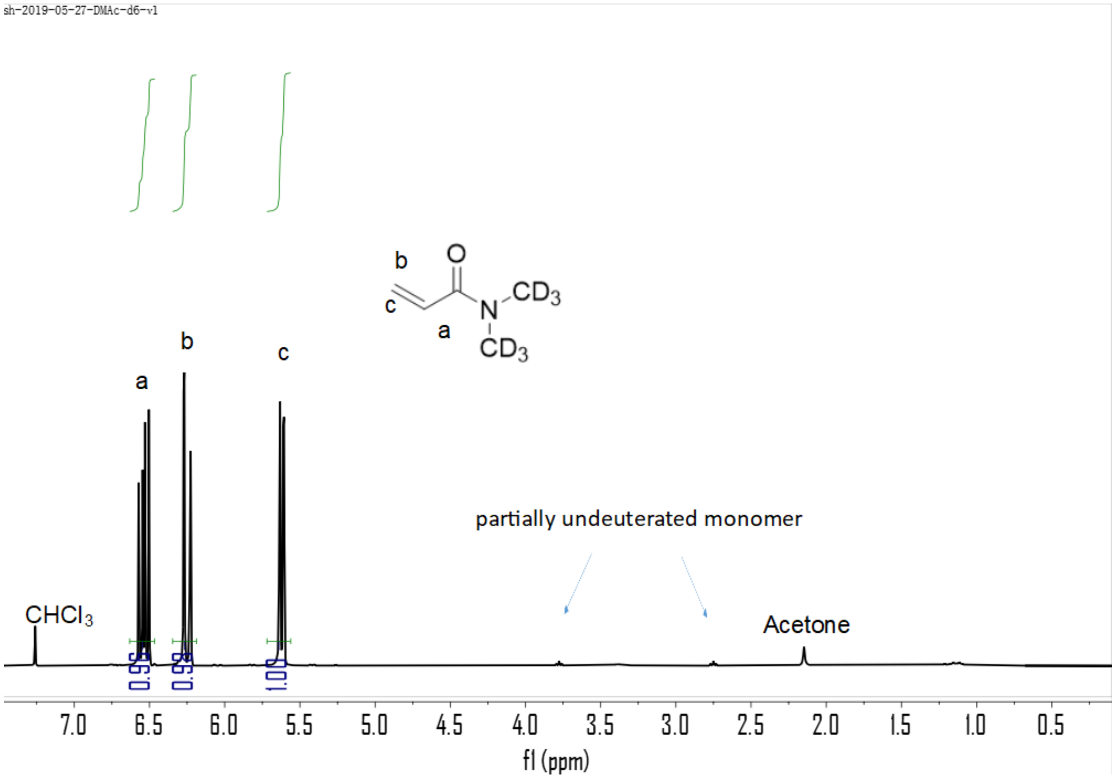


**Supplementary Figure 13:** $^1$H NMR spectrum of **DMAc($D_6$)** ($CDCl_3$).

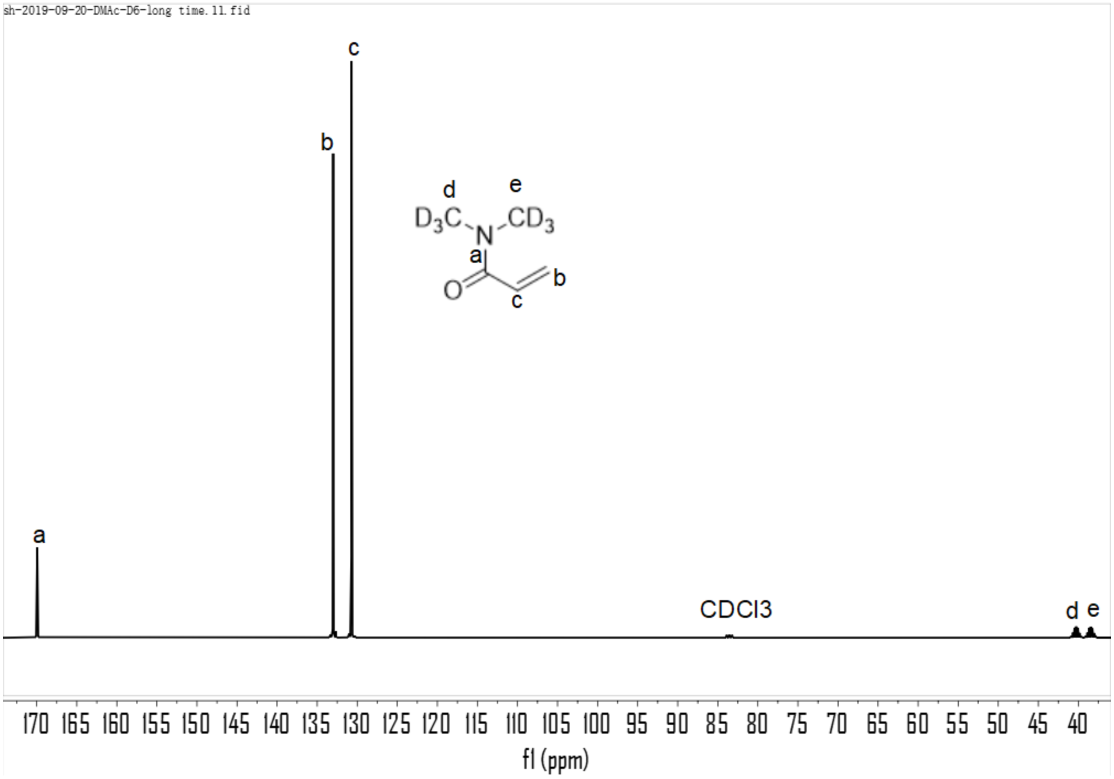


**Supplementary Figure 14:** $^{13}$C NMR spectrum of **DMAc($D_6$)** ($CDCl_3$).

**RAFT polymerization of DMAc($D_6$). 4/8-CTA** (0.100 g, 0.126 mmol, 1eq), DMSO (7.5 mL) and **DMAc($D_6$)** (0.582 g, 5.54 mmol, 44 eq) were introduced in a Schlenk flask. In order to obtain a homogeneous solution, the mixture was heated to 75 $^{o}$C and was degassed by bubbling argon for 30 min. AIBN (2.1 mg, 0.013 mmol, 0.1 eq) was introduced into the solution under argon, and the mixture was degassed for another 5 min. Samples were withdrawn periodically from the reaction mixture to determine conversion by $^{1}$H NMR. After 74% conversion, the Schlenk was opened to air and cooled down to room temperature. The product was precipitated in about 100 mL of DE, filtered and was dissolved in water to perform dialysis with a 1 kDa membrane. A light-yellow powder (400 mg, 75%) was obtained by freeze-drying the dialyzed solution.

$^{1}$H NMR (400 MHz, DMSO-$d_6$) $\delta$5.80-5.63 (m, -N**H**, 6H), 4.04-3.85 (m, -OC$\mathbf{H_2}$-, 2H), 3.30-2.89 (m, -C$\mathbf{H_2}$NH-, 12H), 2.65-2.12 (m, -CHC$\mathbf{H_2}$S-, -C**H**(C=O)N($CD_3$)$_2$), 1.96-1.84 (br, -C**H**($CH_3$)$_2$, 1H), 1.75-0.93 (m, -C$\mathbf{H_2}$CH(C=O)N($CD_3$)$_2$, -$CH_2$C**H**(C$\mathbf{H_2}$$CH_3$)(C$\mathbf{H_2}$)$_3$$CH_3$, -C(C$\mathbf{H_3}$)$_2$, -O$CH_2$(C$\mathbf{H_2}$)$_4$-, -NH(C=O)NH$CH_2$(C$\mathbf{H_2}$)$_6$-, -NH(C=O)NH$CH_2$(C$\mathbf{H_2}$)$_2$-, -CH(C$\mathbf{H_3}$)$_2$, 115H), 0.92-0.78 (m, -$CH_2$C$\mathbf{H_3}$, 6H). See Supplementary Figure 15.

SEC: see Supplementary Figure 21 and Supplementary Table 1.

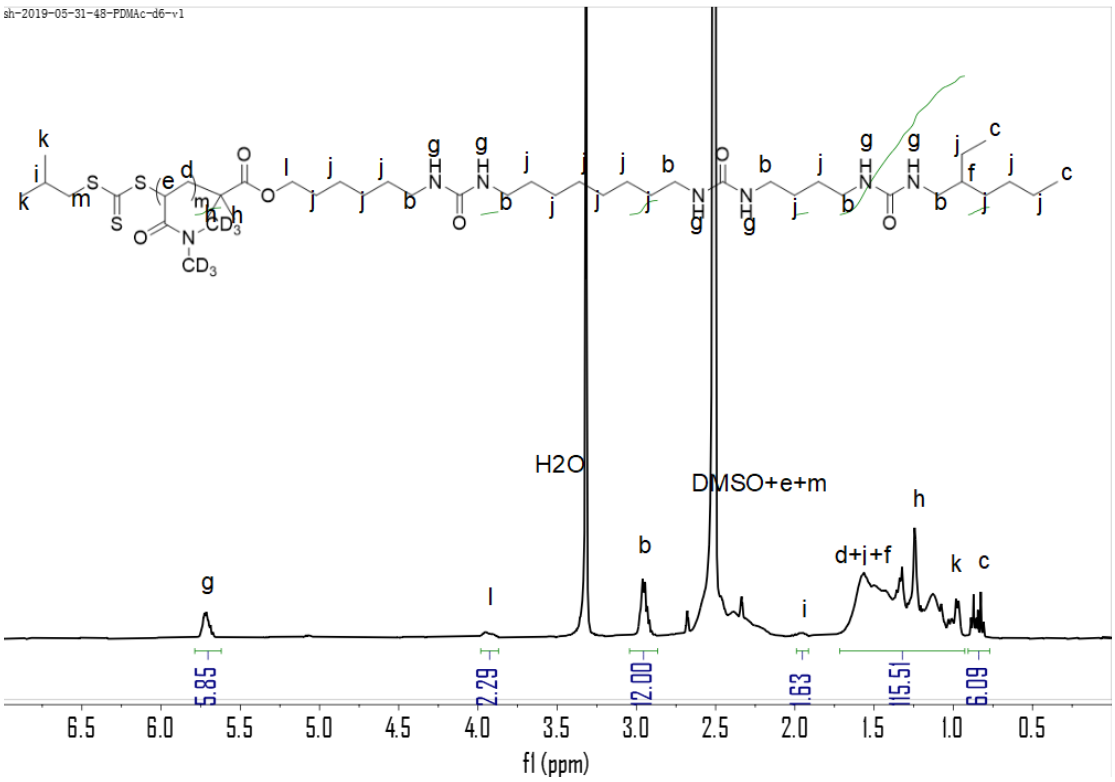


**Supplementary Figure 15:** $^1$H NMR spectrum of **4/8-PDMAc(D$_6$)** (DMSO-d$_6$).

## Synthesis of 4/8-PCEA

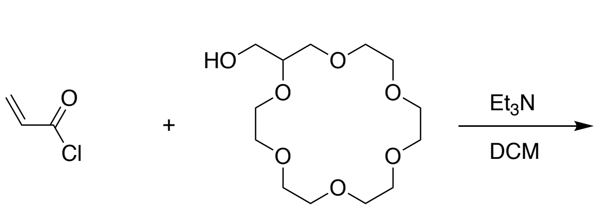


**Supplementary Figure 16:** Synthesis of **CEA**.

**18-crown-6 acrylate (CEA).** Triethylamine (1.5 mL, 10.7 mmol, 1.5eq) was added into 1-hydroxymethyl-18-crown-6 solution (2 g, 6.8mmol, 1eq) in 60 mL DCM) under argon at 0 °C. Acryloyl chloride (0.81 mL, 10.2 mmol, 1.5 eq) in 10 mL DCM was added slowly (10 mL $h^{-1}$). After stirring under argon at room temperature overnight, the mixture was filtered to remove the solid. After evaporating the solvent from the filtrate, the crude product was purified by $Al_2O_3$ column chromatography (ethyl acetate as eluent). 1.853 g (78%) colorless liquid was obtained.

$^1H$ NMR (400 MHz, $CDCl_3$) $\delta$ 6.40 (dd, $J$ = 17.3, 1.4 Hz, CH=C**H**(c)H, 1H), 6.12 (dd, $J$ = 17.3, 10.4 Hz, C**H**=$CH_2$, 1H), 5.82 (dd, $J$ = 10.4, 1.4 Hz, CH=CH**H**(t), 1H), 4.34-4.17 (m, -$CO_2C\mathbf{H}_2$CH-, 2H), 3.87-3.83 (m, -$CH_2$C**H**O-, 1H), 3.82-3.75 (m, -$CH_2OC\mathbf{H}_2$CH-, 2H), 3.73-3.61 (m, -$OC\mathbf{H}_2C\mathbf{H}_2$O-, 20H). See Supplementary Figure 17.

$^{13}C$ NMR (100 MHz, $CDCl_3$) $\delta$ 165.98, 130.88, 128.30, 77.17, 71.11, 71.00, 70.98, 70.89, 70.82, 70.74, 70.69, 70.02, 64.10. See Supplementary Figure 18.

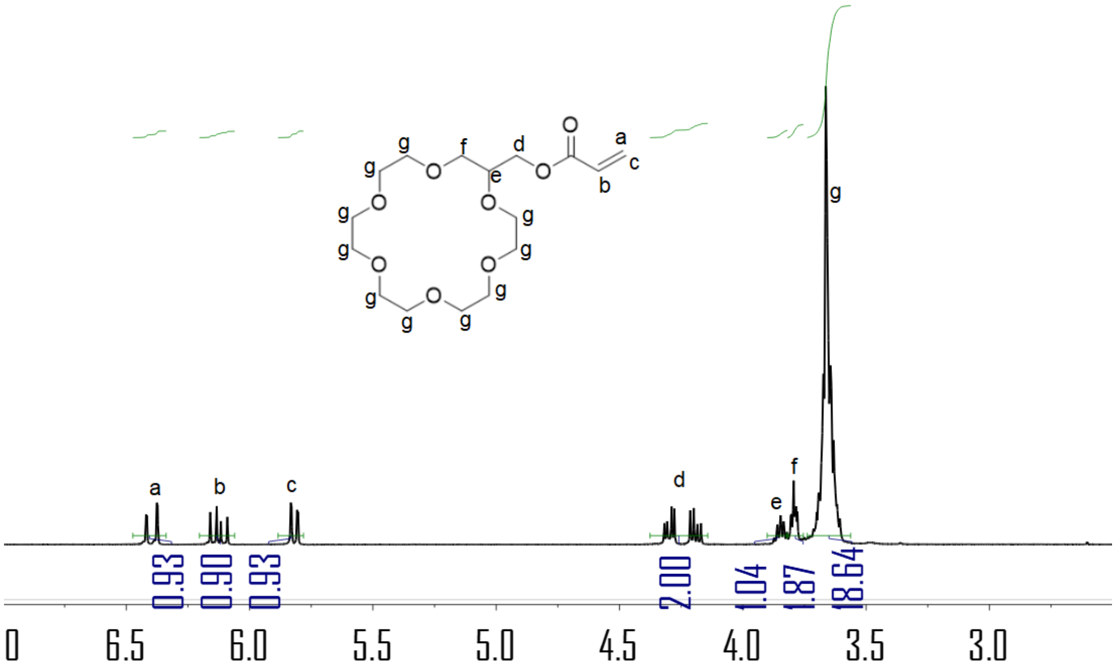


**Supplementary Figure 17:** $^1H$ NMR spectrum of **CEA** ($CDCl_3$).

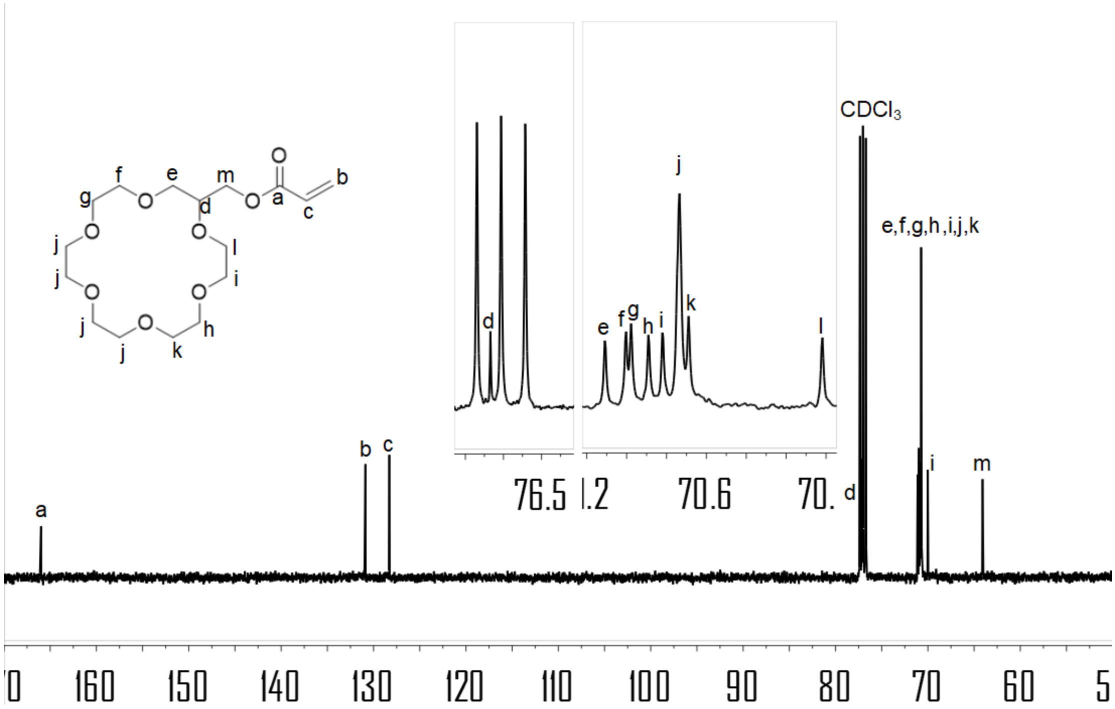


**Supplementary Figure 18:** $^{13}$C NMR spectrum of **CEA** ($CDCl_3$).

**RAFT polymerization of CEA. 4/8-CTA** (0.034 g, 0.043 mmol, 1eq), DMSO (1.5 mL) and **CEA** (0.600 g, 1.72 mmol, 40 eq) were introduced in a Schlenk flask. In order to obtain a homogeneous solution, the mixture was heated to 75 °C and was degassed by bubbling argon for 30 min. AIBN (1.1 mg, 0.0067 mmol, 0.16 eq) was introduced into the solution under argon, and the mixture was degassed for another 5 min. Samples were withdrawn periodically from the reaction mixture to determine conversion by $^{1}$H NMR. After 72% conversion, the Schlenk was opened to air and cooled down to room temperature. The product was precipitated in about 100 mL of DE, filtered and was dissolved in water to perform dialysis with a 1 kDa membrane. A light-yellow solid (300 mg, 67%) was obtained by freeze drying the dialyzed solution.

$^{1}$H NMR (400 MHz, DMSO-$d_6$) $\delta$ 5.78-5.63 (m, -N**H**, 6H), 4.31-3.81 (m, -COOC**H**$_2$-, -COOCH$_2$C**H**-, 77H), 3.79-3.40 (m, -C**H**$_2$OC**H**$_2$-, 550H), 3.04-2.88 (m, -C**H**$_2$NH-, 12H), 2.42-2.08 (m, -CHC**H**$_2$S-, -C**H**CH$_2$-, 29H), 2.06-1.16 (m, (CH$_3$)$_2$C**H**CH$_2$-, -CHC**H**$_2$-, -CH2C**H**CH2-, -CH$_2$C**H**$_2$CH$_2$-, CH$_3$C**H**$_2$-, -CHC**H**$_2$-, 84H), 1.13-1.03 (m, (C**H**$_3$)C-, 6H), 1.01-0.94 (m, (C**H**$_3$)CH-, 6H), 0.91-0.78 (m, C**H**$_3$CH$_2$-, 6H). See Supplementary Figure 19.

SEC: see Supplementary Figure 21 and Supplementary Table 1.

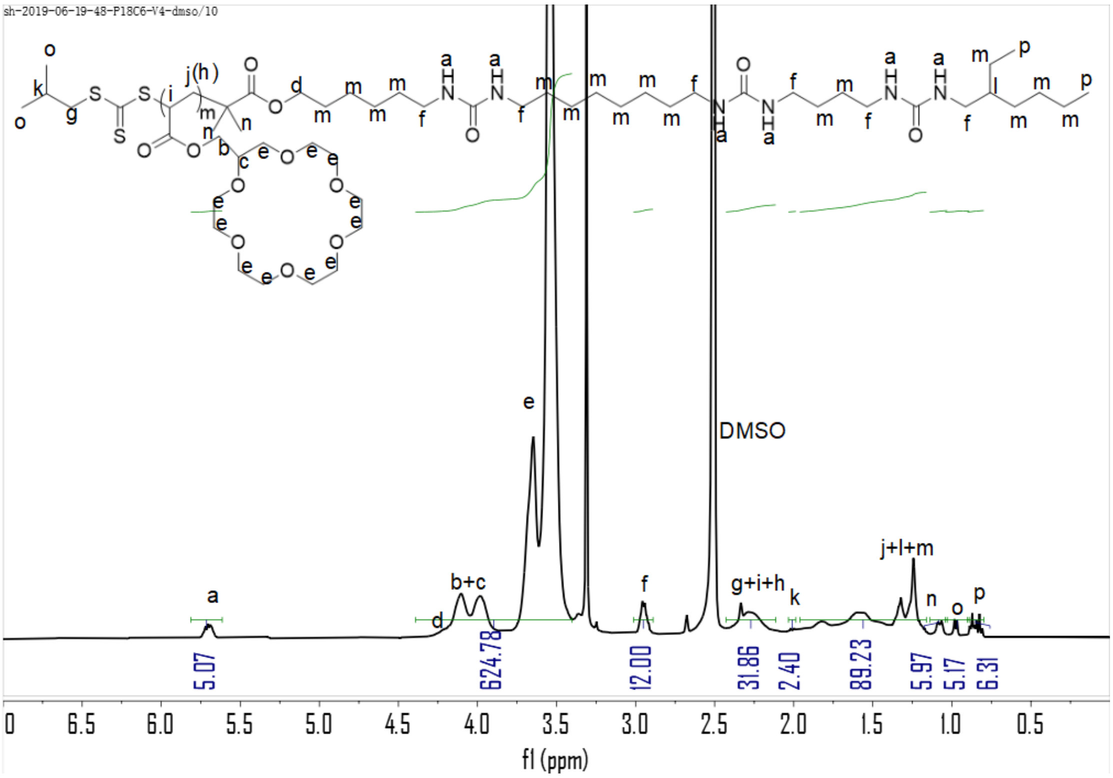

**Supplementary Figure 19:** $^1$H NMR spectrum of **4/8-PCEA** (DMSO-$d_6$).

**RAFT polymerization of *n*-butylacrylate (*n*BA). 4/8-CTA** (0.101 g, 0.128 mmol, 1eq), DMSO/dioxane (1/1 v/v) (7.5 mL) and *n*BA (0.830 g, 551 mmol, 42 eq) were introduced in a Schlenk flask. In order to obtain a homogeneous solution, the mixture was heated to 75 °C and was degassed by bubbling argon for 30 min. AIBN (2.1 mg, 0.013 mmol, 0.1 eq) was introduced into the solution under flowing argon, and the mixture was degassed for another 5 min. Samples were withdrawn periodically from the reaction mixture to determine conversion by $^1$H NMR. After 77% conversion, the Schlenk was opened to air and cooled down to room temperature. The product was precipitated in about 100 mL of a cold mixture of MeOH/$H_2O$=7/3 and centrifuged. The lower layer was dissolved in DCM and precipitated again (twice), and the lower layer was finally dried under vacuum. A light-yellow rubbery solid (450 mg, 60%) was obtained.

$^1$H NMR (400 MHz, DMSO-$d_6$/$CDCl_3$, 1/9 v/v) $\delta$ 5.75 (m, -N**H**, 6H), 3.96 (m, -(C=O)OC**H**$_2$-, 69H), 3.24 (m, -SC**H**$_2$-, 2H), 2.97 (m, -C**H**$_2$NH-, 12H), 2.20 (m, -C**H**(C=O)O-, 32H), 1.96-1.68 (m, -(C=O)CH(C**H**$_3$)$_2$, $(CH_3)_2$C**H**-, -$(CH_2)_2$C**H**-, 8H), 1.70-1.12 (m, -C**H**$_2$CH(C=O)OCH$_2$C**H**$_2$C**H**$_2$CH$_3$, -NHCH$_2$(C**H**$_2$)$_2$-, -NHCH$_2$(C**H**$_2$)$_4$-, -NHCH$_2$(C**H**$_2$)$_6$-, CH$_3$(C**H**$_2$)$_3$-, CH$_3$C**H**$_2$CH-, 226H), 1.12-1.01 (m, (C**H**$_3$)$_2$CH-, 6H), 1.01-0.79 (m, C**H**$_3$CH$_2$-, 107H). See Supplementary Figure 20.

SEC: see Supplementary Figure 21 and Supplementary Table 1.

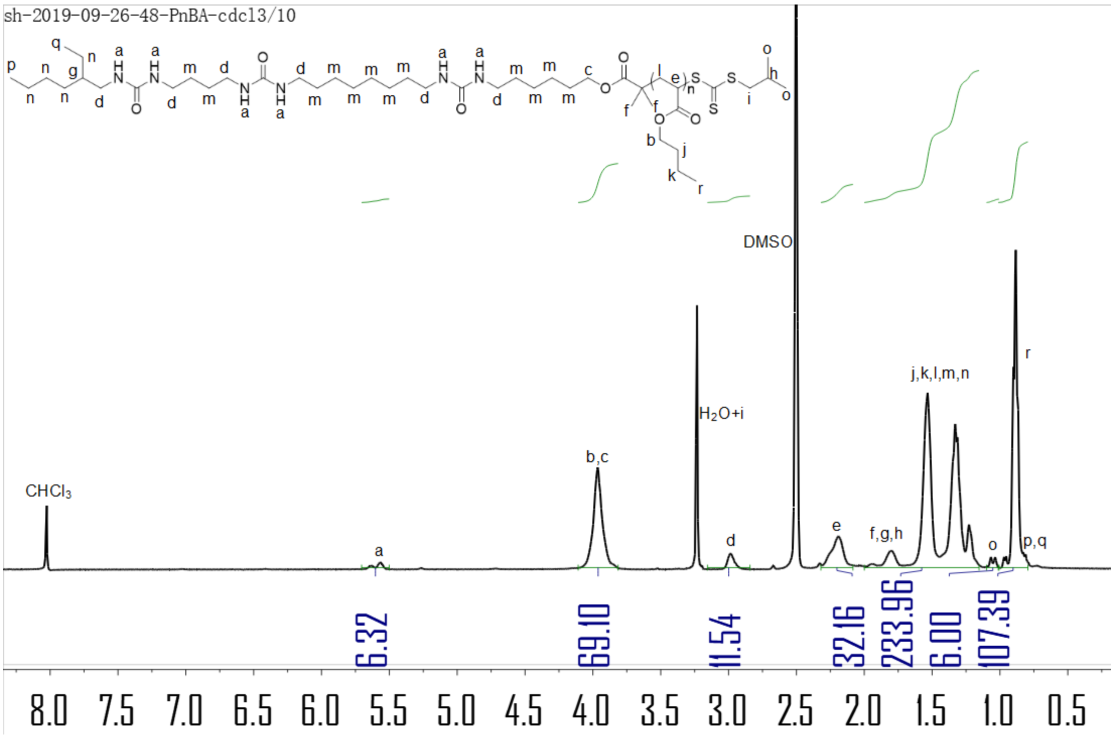


**Supplementary Figure 20:** $^{1}$H NMR spectrum of **4/8-P*n*BA** (DMSO-d6/CDCl3, 1/9 v/v).

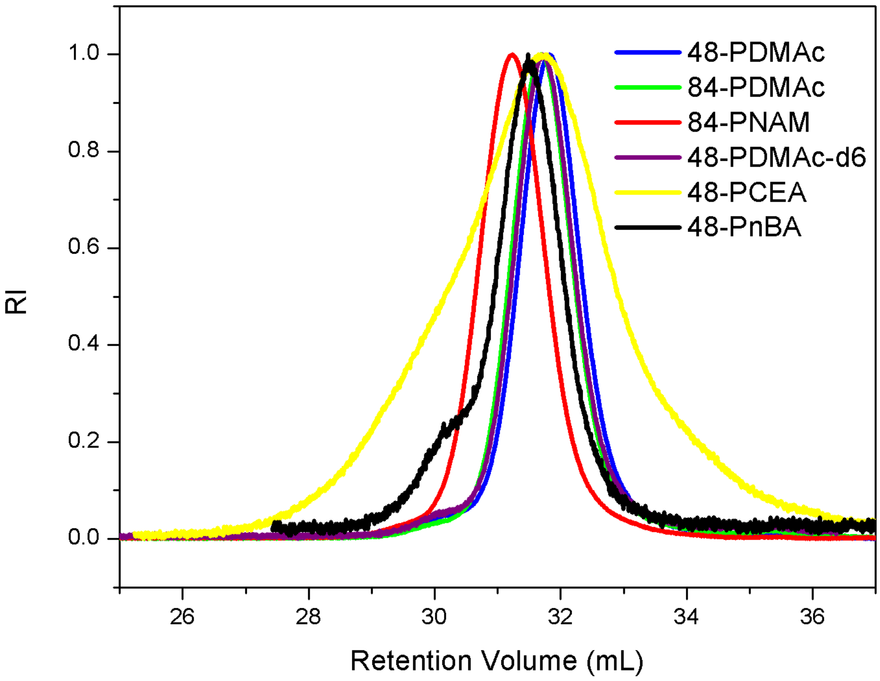


**Supplementary Figure 21:** SEC of **4/8-PDMAc**, **8/4-PDMAc**, **8/4-PNAM**, **4/8-PDMAc(D$_6$)**, **4/8-PCEA** and **4/8-P*n*BA**.

**Supplementary Table 1**. Summary of the polymer molar masses.

| name | RAFT agent | monomer | [M]/[CTA] /[initiator] | conv. (%) [a] | $M_{n,th}$ (g mol$^{-1}$) [b] | $M_{n,NMR}$ (g mol$^{-1}$) [c] | $M_{n,SEC}$ (g mol$^{-1}$) [d] | $Đ$ [d] |
|---|---|---|---|---|---|---|---|---|
| **4/8-PDMAc** | 4/8-CTA | DMAc | 44/1/0.1 | 72 | 3900 | 3900 | 5300 | 1.11 |
| **8/4-PDMAc** | 8/4-CTA | DMAc | 43/1/0.1 | 74 | 3900 | 3800 | 5600 | 1.11 |
| **8/4-PNAM** | 8/4-CTA | NAM | 44/1/0.1 | 89 | 6300 | 7300 | 7200 | 1.10 |
| **4/8-PDMAc($D_6$)** | 4/8-CTA | DMAc | 44/1/0.1 | 74 | 4200 | 4400 | 5600 | 1.10 |
| **4/8-PCEA** | 4/8-CTA | CEA | 40/1/0.1 | 72 | 11000 | 9500 | 6300 | 1.66 |
| **4/8-P*n*BA** | 4/8-CTA | *n*BA | 42/1/0.1 | 77 | 4900 | 5300 | 9400 | 1.11 |

[a] Conversion was calculated by $^1$H NMR.
[b] Theoretical $M_n$ at the conversion where the reaction was stopped.
[c] Calculated based on the $^1$H NMR of the final polymer.
[d] Determined by SEC in DMF (+ 1 g L$^{-1}$ LiBr) using PMMA standards and refractive index detection.

## 1.4. JANUS NANORODS PREPARATION

### 1.4.1 General procedure for all polymers except P*n*BA

**Samples for SLS, cryo-TEM and NMR (relaxation).** The polymers were independently dissolved in DMSO at a concentration of 100 g $L^{-1}$ and mixed in a 1/1 molar proportion. Water was added into the stirred mixture with a syringe pump at a rate of 0.5 mL $h^{-1}$ to obtain a final total polymer concentration of 1 g $L^{-1}$ in DMSO/water (1/99 v/v). Deuterated solvents were used in the case of NMR. To reach higher concentrations, it is possible to evaporate part of the water (see part 7.1) or to freeze-dry the particles (see below) after removal of DMSO by dialysis.

**Solid powder.** DMSO was removed from the solution at 1 g $L^{-1}$ in DMSO/water (1/99) by dialysis against water for 3 days using a dialysis membrane (MWCO = 1 kDa) and the solution was freeze-dried. The yield is almost quantitative (80-90%), loss of material being only due to transfer operations. The powder can then be dispersed at any concentration in water by manual shaking of the vial.

### 1.4.2 Procedure for P*n*BA

**Samples for SLS. 4/8-P*n*BA** and **8/4-PDMAc** were independently dissolved in ethanol at 50 °C at a concentration of 100 g $L^{-1}$ and mixed in a 1/1 molar proportion. Dioxane was added into the stirred mixture (maintained at 50 °C) with a syringe pump at a rate of 0.5 mL $h^{-1}$ to obtain a final polymer concentration of 1 g $L^{-1}$ in ethanol/dioxane (1/99 v/v).

# 2. SUPPLEMENTARY NOTES

## 2.1. JANUS NANORODS CHARACTERIZATIONS

**Static light scattering (SLS).** Measurements were conducted using an ALV-CGS3 system operating with a vertically polarized laser at a wavelength λ=633 nm. See reference[2] for details. The measurements were done at 20 °C over a range of scattering wave vectors ($q = 4\pi n.\sin(\theta/2)/\lambda$, with $\theta$ the angle of observation and $n$ the refractive index of the solvent) varying from 2.7 $10^6$ $m^{-1}$ to 2.6 $10^7$ $m^{-1}$. The DMSO (resp. ethanol) solutions of the polymers were filtered over 0.45 µm GHP Acrodisc filters before water (resp. dioxane) addition. Measurements of the specific refractive index increment (dn/dc) of PDMAc (5200 g $mol^{-1}$) and PNAM (6900 g $mol^{-1}$) were performed in water at 20 °C on a differential refractometer, Optilab rEX Wyatt-822, equipped with a laser light source ($\lambda$ = 633 nm). dn/dc = 0.17 mL $g^{-1}$ was determined both for PDMAc and for PNAM. These values were used to normalize the SLS data for all polymers no matter the method of preparation.

The Rayleigh ratio, $R_\theta$, of the solution, corresponding to one single population of scatterers, was determined following Supplementary Equation 1.

$$R_\theta = \frac{I_{\text{solution}}(\theta) - I_{\text{solvent}}(\theta)}{I_{\text{toluene}}(\theta)} \cdot \left(\frac{n_{\text{solvent}}}{n_{\text{toluene}}}\right)^2 \cdot R_{\text{toluene}} \qquad (1)$$

with $I_{\text{solution}}$, $I_{\text{solvent}}$, $I_{\text{toluene}}$ the average intensities scattered respectively by the solution, the solvent and the reference (toluene), $n_{\text{solvent}}$ = 1.333 (water) and $n_{\text{toluene}}$ = 1.496 the respective refractive indexes of the solvent and of toluene and $R_{\text{toluene}} = 1.35\times10^{-5}$ $cm^{-1}$ the Rayleigh ratio of toluene for a wavelength $\lambda$ = 633 nm. For water/DMSO (99/1 vol/vol) solutions, $n_{\text{solvent}}$ was taken equal to $n_{\text{water}}$.

If self-sorting occurred, the raw scattered intensity for the mixture should be equal to the sum of the scattered intensities for the pure component solutions (measured at the same respective concentrations as in the mixture). In fact, the raw scattered intensity measured for the mixtures of **4/8-PDMAc** and **8/4-PNAM** (Supplementary Figure 22) and **4/8-P*n*BA** and **8/4-PDMAc** (Supplementary Figure 23) are much higher than the calculated sum of the scattered intensities of the pure component solutions, therefore proving co-assembly of the two components.

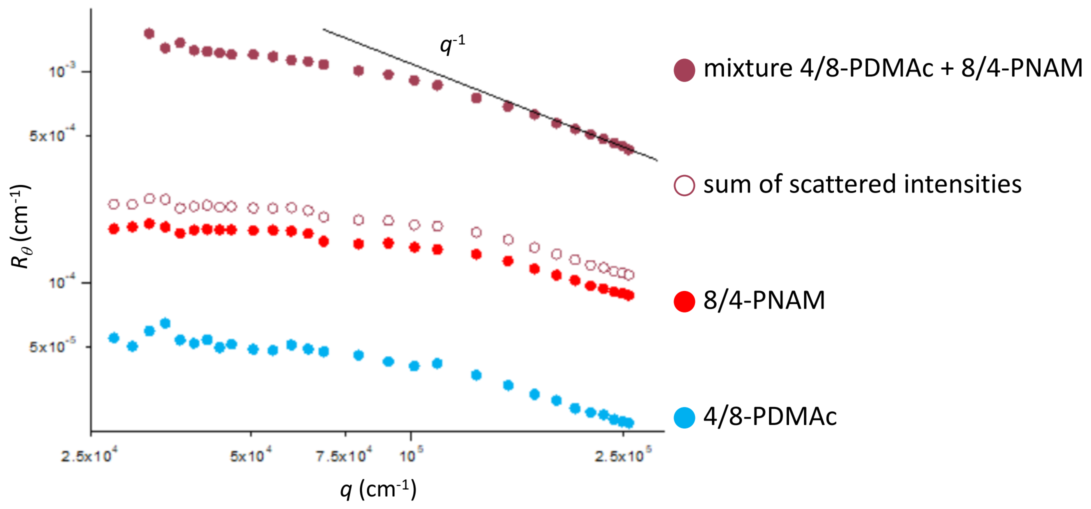


**Supplementary Figure 22:** Light scattering raw intensity for solutions of either **4/8-PDMAc** (0.36 g L$^{-1}$), **8/4-PNAM** (0.64 g L$^{-1}$) or their equimolar mixture: **4/8-PDMAc** + **8/4-PNAM** (0.36 g L$^{-1}$ and 0.64 g L$^{-1}$ final concentrations) in water/DMSO (99/1). The much higher intensity for the mixture compared to the sum of intensities scattered by each individual polymer solution, proves the co-assembly. The beginning of a $q^{-1}$ dependence of the scattered intensity at high scattering vector ($q$) suggests the formation of rod-like objects.

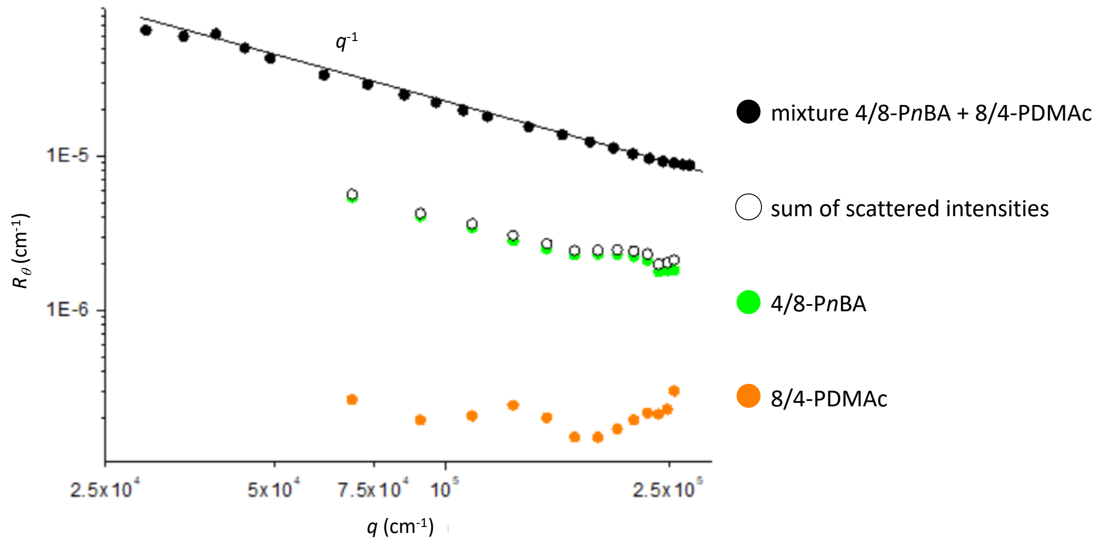


**Supplementary Figure 23:** Light scattering raw intensity for solutions of either **4/8-P*n*BA** (0.6 g L$^{-1}$), **8/4-PDMAc** (0.4 g L$^{-1}$) or their equimolar mixture: **4/8-P*n*BA** + **8/4-PDMAc** (0.6 g L$^{-1}$ and 0.4 g L$^{-1}$ final concentrations) in dioxane/ethanol (99/1). The much higher intensity for the mixture compared to the sum of intensities scattered by each individual polymer solution, proves the co-assembly. The $q^{-1}$ dependence of the scattered intensity proves the formation of rod-like objects.

**Small angle neutron scattering (SANS).** Small-angle neutron scattering measurements were made at the LLB (Saclay, France) on the PA20 instrument, at two distance-wavelength combinations to cover the 4.6 $10^{-3}$ to 0.3 $Å^{-1}$ $q$-range, where the scattering vector $q$ is defined as usual, assuming elastic scattering ($q = (4\pi/\lambda)\sin(\theta/2)$, where $\theta$ is the angle between incident and scattered beam). Data were corrected for the empty cell signal and the solute and solvent incoherent background. A light water standard was used to normalize the scattered intensities to $cm^{-1}$ units. The freeze-dried Janus nanorod samples were directly dissolved in $D_2O$ (10 g $L^{-1}$).

The SANS data were fitted using the open source SASView software (http://www.sasview.org/). A model of infinitely long and rigid cylinders with an elliptical cross-section and a uniform contrast was used to fit the data for $q < 0.07$ $Å^{-1}$ (Fig. 2c). The discrepancy between the model and the experiment at high $q$ is due to the fact that the solvated polymer chains at the surface of the objects are not explicitly taken into account in this model. The scattering length density (SLD) for PDMAc and $D_2O$ were calculated from the atomic bound coherent scattering lengths ($SLD_{PDMAc} = 0.94$ $10^{-6}$ $Å^{-2}$ and $SLD_{D2O} = 6.37$ $10^{-6}$ $Å^{-2}$).

**8/4-PDMAc** + **4/8-PDMAc**. The major and minor diameters of the cross-section were adjusted to fit the data (Supplementary Figure 24). The obtained values ($d_{minor} = 7.6$ nm and $d_{major} = 12$ nm) are consistent with other PDMAc nanorods.[3] In the case of cylindrical objects, the intensity at low $q$ can be expressed as

$$(qI)_{q\to 0} = \pi c \left(\frac{SLD_{PDMAc} - SLD_{D2O}}{d}\right)^2 M_L \qquad (2)$$

Where $c$ is the polymer concentration ($10^{-2}$ g $cm^{-3}$), $d$ is the PDMAc density (1.094 g $cm^{-3}$) and $M_L$ is the molar mass per unit length of the cylinder. The measured intensity at low $q$ yields a value for $M_L = 12000$ g $mol^{-1}$ $nm^{-1}$. Two consecutive ureas interacting through hydrogen bonds in a 1D structure are separated by an intermolecular distance of 0.46 nm.[4] Therefore, the number of molecules in the cross-section of the cylinders can be estimated as $n_{cs} = M_L \times 0.46 / M_{w,unimer} = 1.3$. This value is consistent with the expected value of 1.

**8/4-PDMAc** + **4/8-PDMAc($D_6$)**. The minor diameter of the cross-section was imposed to be the same as for **8/4-PDMAc** + **4/8-PDMAc** ($d_{minor} = 7.6$ nm) and the major diameter was adjusted to fit the data (Supplementary Figure 24). The obtained value ($d_{major} = 9.4$ nm) is consistent with a Janus structure where the deuterated cylinder half has a very low contrast (Supplementary Figure 25).

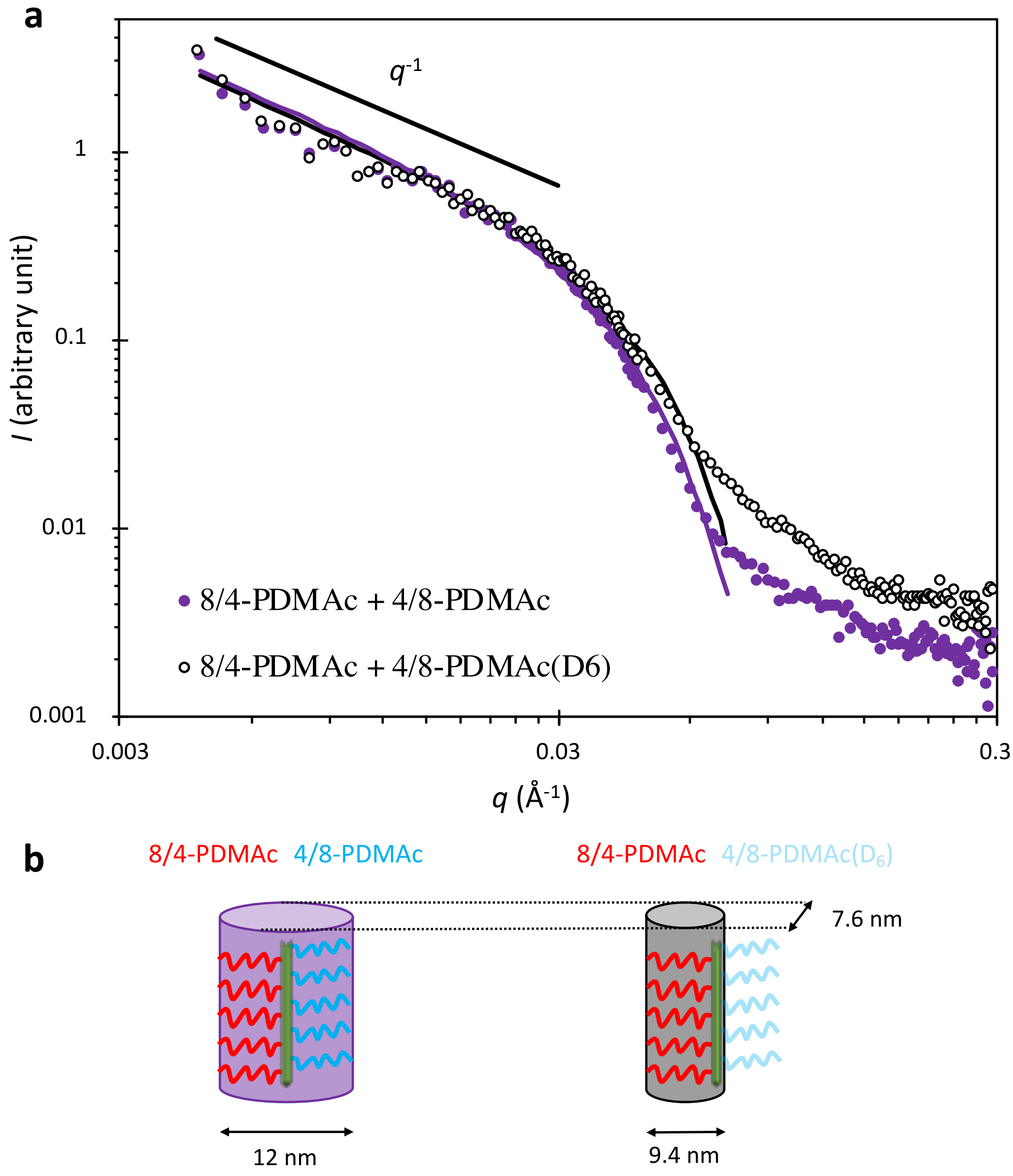


**Supplementary Figure 24: a**, Normalized SANS data of (**4/8-PDMAc(D$_6$)** + **8/4-PDMAc**) and (**4/8-PDMAc** + **8/4-PDMAc**) co-assemblies (freeze-dried particles, 10 g L$^{-1}$ in $D_2O$). The data of Fig. 2b was normalized to highlight the distinct dimensions of the cross-sections. The curves are fits with the form factor of cylinders of elliptical cross-section. **b**, Dimensions of the cross-sections.

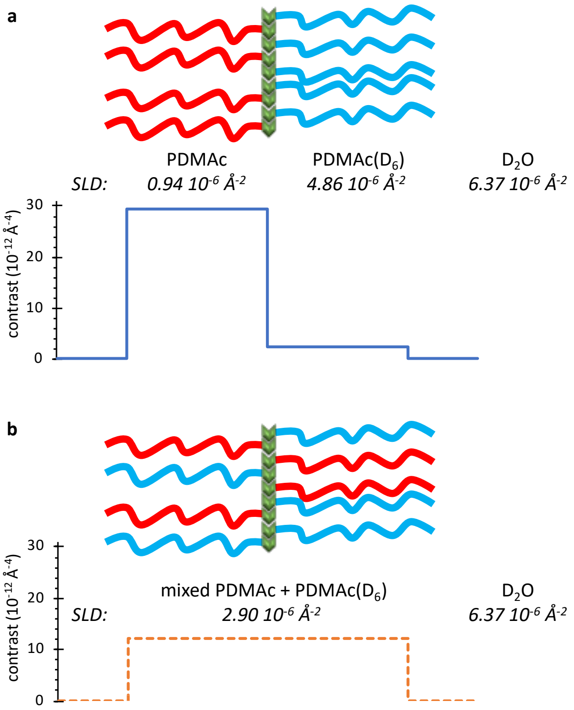


**Supplementary Figure 25:** Calculated contrast for possible co-assemblies formed by (**4/8-PDMAc($D_6$)** + **8/4-PDMAc**) in $D_2O$. The contrast is calculated from the scattering length densities (SLD) as $[SLD_{polymer} - SLD_{D2O}]^2$.
**a**, segregation of deuterated chains. **b**, random mixing of deuterated chains.

**$^1$H Pulsed-Field-Gradient (PFG) NMR measurements.** The $^1$H pulsed-field-gradient NMR measurements were performed at a regulated temperature of 23 °C with a Bruker Avance III NMR spectrometer operating at a $^1$H Larmor frequency of 300.13 MHz and a *z*-gradient 5 mm diffusion NMR probe head (Diff30L, 30 A, maximum strength of 1200 G cm$^{-1}$). The spin echo attenuation resulting from diffusion was monitored as a function of the gradient strength, *g*, involved in the pulsed-field-gradient stimulated echo (STE) pulse sequence with LED and bipolar gradients. The $^1$H 90° pulse length was set to 9.6 μs and the recycle delay to 3 s. A trapezoidal shape was used for the gradient pulses and the effective gradient pulse length $\delta$ was equal to 600 μs. The diffusion time between gradient pulses, $\Delta$, was fixed at 150 ms. The freeze-dried Janus nanorod sample was directly dissolved in $D_2O$ (5 g L$^{-1}$).

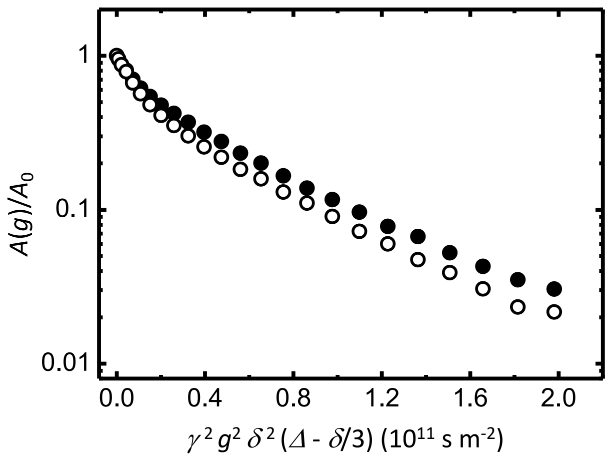


**Supplementary Figure 26:** Evolution of the stimulated echo amplitude *A* versus $\gamma^2 g^2 \delta^2 (\Delta - \delta/3)$, in which *g* denotes the gradient strength. $\gamma$ corresponds to the $^1$H gyromagnetic ratio; $\Delta$, the diffusion time; $\delta$, the effective gradient pulse length and $A_0$, the echo amplitude obtained for $g = 0$ G cm$^{-1}$. These decays were monitored for the protons for the -O-$CH_2$-$CH_2$-N- groups of PNAM (solid circles) and for the -N$(CH_3)_2$ methyl protons of PDMAc (hollow circles), using an equimolar mixture (**4/8-PDMAc + 8/4-PNAM**) (freeze-dried particles, 5 g L$^{-1}$ in $D_2O$).

**2D $^1$H NOESY.** The 2D $^1$H NOESY experiments were carried out with a Bruker Avance III NMR spectrometer operating at a $^1$H Larmor frequency of 300.13 MHz and a BBFO *z* gradient 5 mm probe head. The $^1$H 90° pulse length was equal to 11.1 μs, the mixing time $t_m$, to 500 ms and the recycle delay to 2 s. Phase-sensitive 2D time domains were acquired and processed following the States-TPPI mode with 1024 slices recorded along the $t_1$ dimension. The freeze-dried Janus nanorod samples were directly dissolved in $D_2O$ (5 g L$^{-1}$). The measurements were carried out at a regulated temperature of 23 °C.

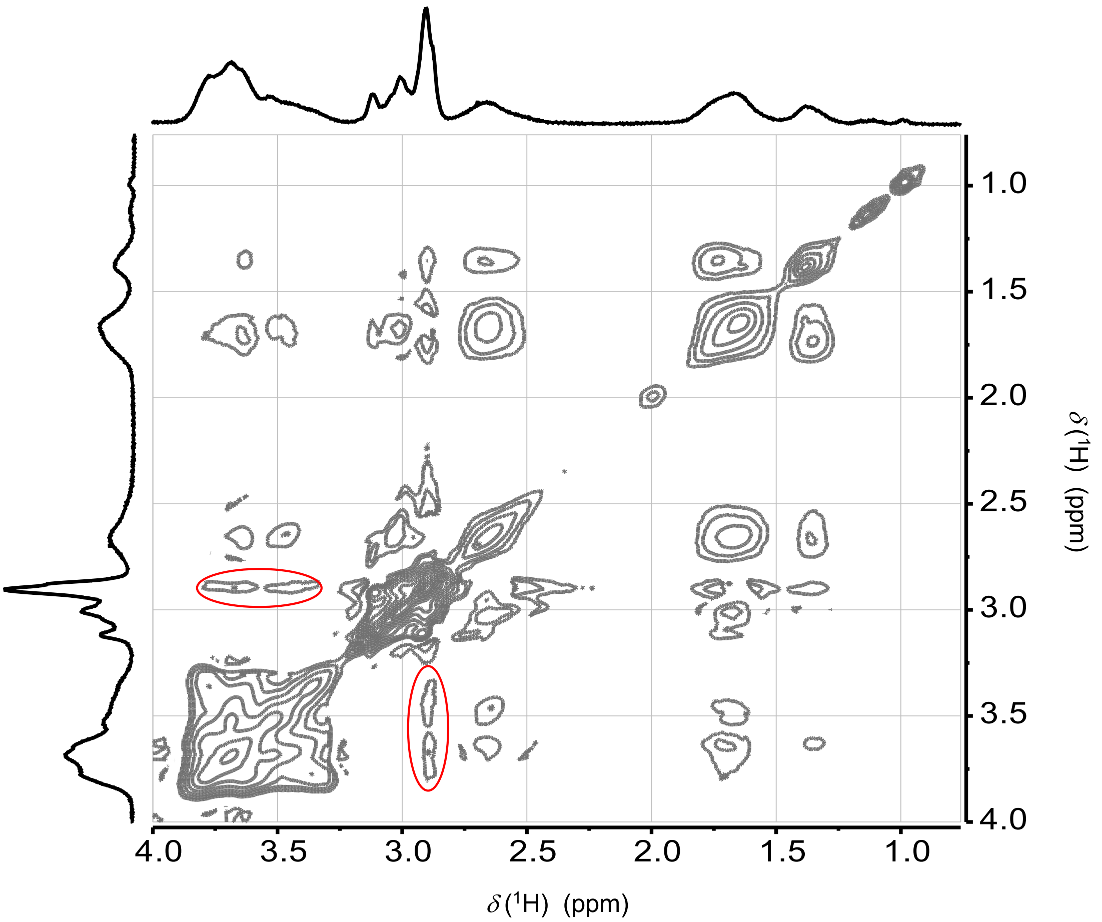


**Supplementary Figure 27:** $^1$H NOESY contour plot for a solution of PDMAc-co-PNAM statistical copolymer in $D_2O$ (freeze-dried particles, 5 g $L^{-1}$). Cross-correlations between the methyl protons of DMAc units and the methylene protons of morpholine units are indicated by red ellipses.

**1D $^1$H Transverse Relaxation Experiments.** $^1$H NMR transverse relaxation functions were determined at a regulated temperature of 25 °C using a Bruker Avance III NMR spectrometer ($^1$H Larmor frequency of 300.13 MHz), equipped with a BBFO *z* gradient 5 mm probe head. The $^1$H transverse relaxation signals were measured by means of the PROJECT pulse sequence (Periodic Refocusing of J Evolution by Coherence Transfer). The $^1$H 90° pulse length was equal to 11.0 μs, the interpulse delay to 0.125 ms and the recycle delay to 3 s. The amplitude of the $^1$H transverse relaxation signals, $A(t)$, was normalized by $A_0$, which corresponds to their value following a single cycle of the PROJECT pulse sequence. The $^1$H $T_2$ relaxation measurements were carried out at a sample concentration of 1 g $L^{-1}$ in $D_2O$ (after dialysis of a $D_2O$/DMSO-$d_6$, 99/1 v/v solution).

**Cryo-TEM.** A drop of the sample solution (1 g $L^{-1}$ in water/DMSO, 99/1 v/v) was deposited on a Quantifoil coated carbon grid. After absorption of the excess solution by filter paper, the grid was flash frozen in liquid ethane and observed at -180 °C on a JEOL JEM-2100 LaB6 microscope operating at 200 kV under low-dose conditions (10 electrons $Å^{-2}$ $s^{-1}$). Digital images were recorded on a 2k x 2k CCD camera, Gatan Ultrascan 1000.

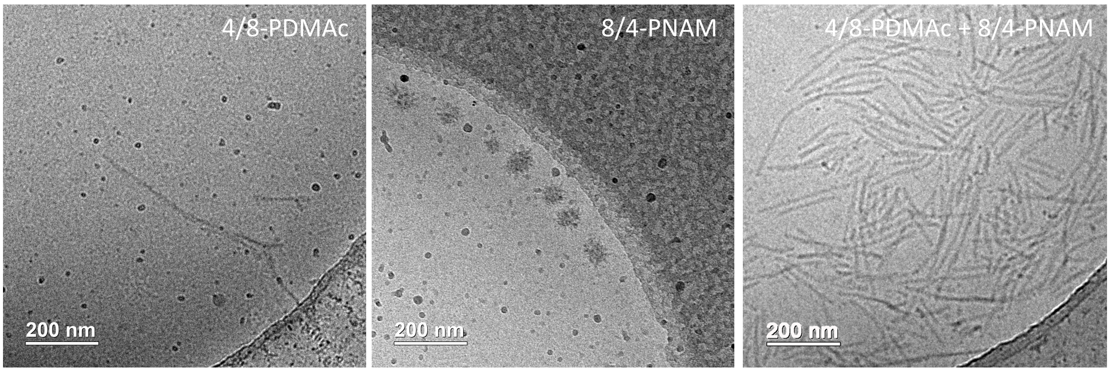


**Supplementary Figure 28:** Cryo-TEM images of **4/8-PDMAc**, **8/4-PNAM** and their equimolar mixture (**4/8-PDMAc** + **8/4-PNAM**). 1 g $L^{-1}$ in water/DMSO (99/1).

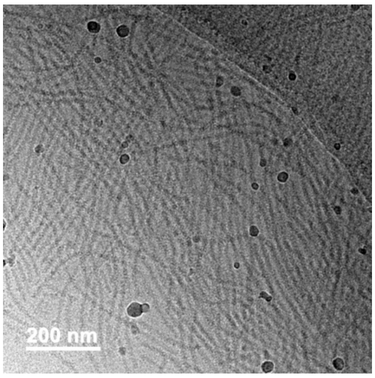


**Supplementary Figure 29:** Cryo-TEM image of Janus nanorods obtained from (**4/8-PDMAc** + **8/4-PNAM**) after dialysis, freeze-drying and dispersion in pure water.

**TEM.** A **4/8-PCEA** 100 g $L^{-1}$ DMSO solution and an **8/4-PDMAc** 100 g $L^{-1}$ DMSO solution were mixed together in equimolar proportions (volume ratio (14/5)). Pure water was added (0.5 mL $h^{-1}$) until a water/DMSO proportion of 99/1 (v/v). DMSO was removed by dialysis against water for 3 days using a dialysis membrane (MWCO = 1 kDa). A silver nitrate solution in water (0.1 mol $L^{-1}$) was added (1 equivalent Ag per crown ether). A drop of the sample solution was deposited on a carbon coated grid, stained with a drop of uranyl acetate aqueous solution (20 g $L^{-1}$), and observed on a JEOL JEM-2100 LaB6 microscope operating at 200 kV. Digital images were recorded on a 2k x 2k CCD camera, Gatan Ultrascan 1000.

The blank sample (Supplementary Figure 31) was prepared in the same manner from **4/8-PDMAc** and **8/4-PDMAc**, and contained the same concentration of silver nitrate.

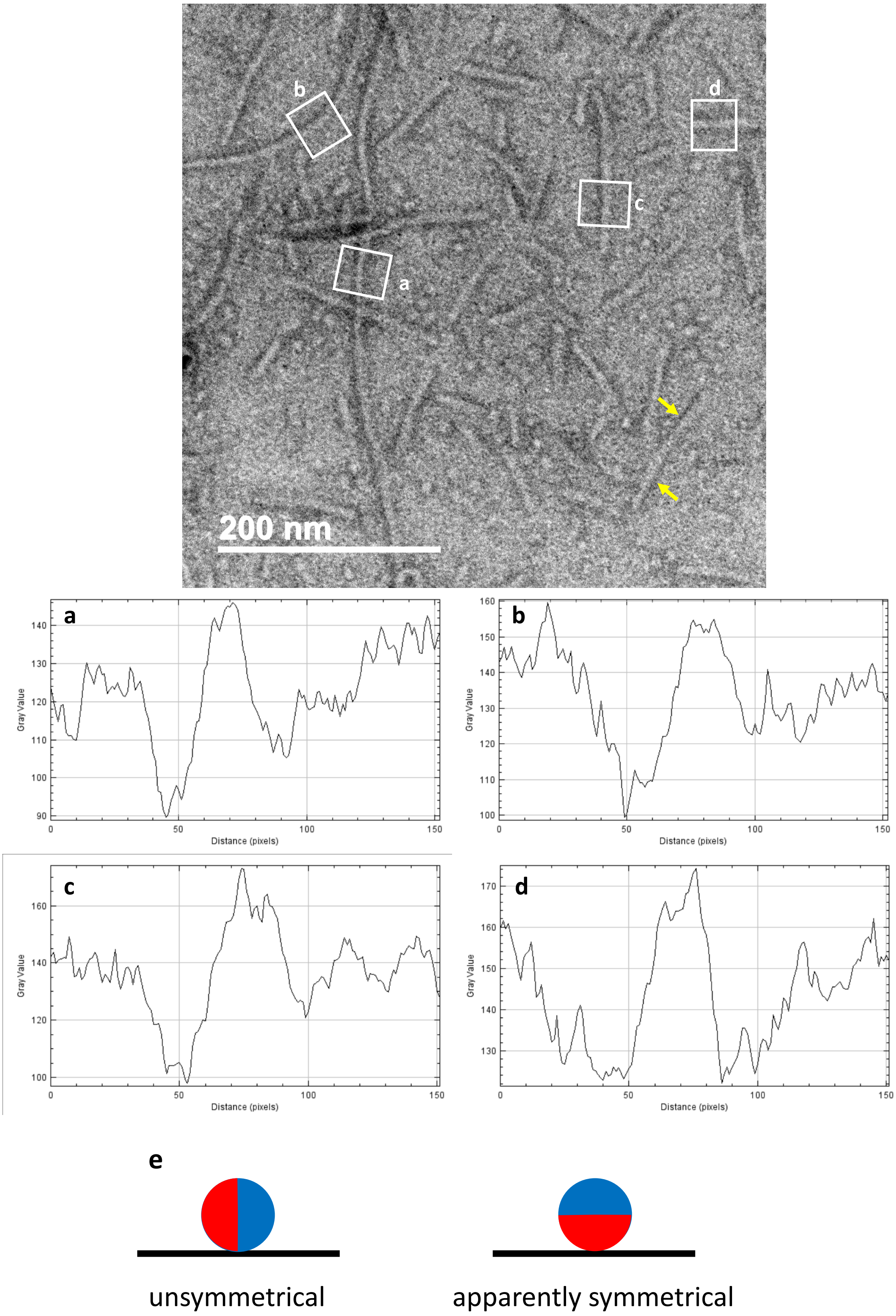


**Supplementary Figure 30:** Non-symmetrical assembly. Top: TEM of (**4/8-PCEA** + **8/4-PDMAc**) equimolar mixture containing $AgNO_3$ (negative staining with uranyl acetate). Yellow arrows highlight contrast inversion along the nanorods axis. Bottom: grey scale analysis of the highlighted areas. **a-c**: unsymmetrical objects. **d**: apparently symmetrical object. **e**: rationalization.

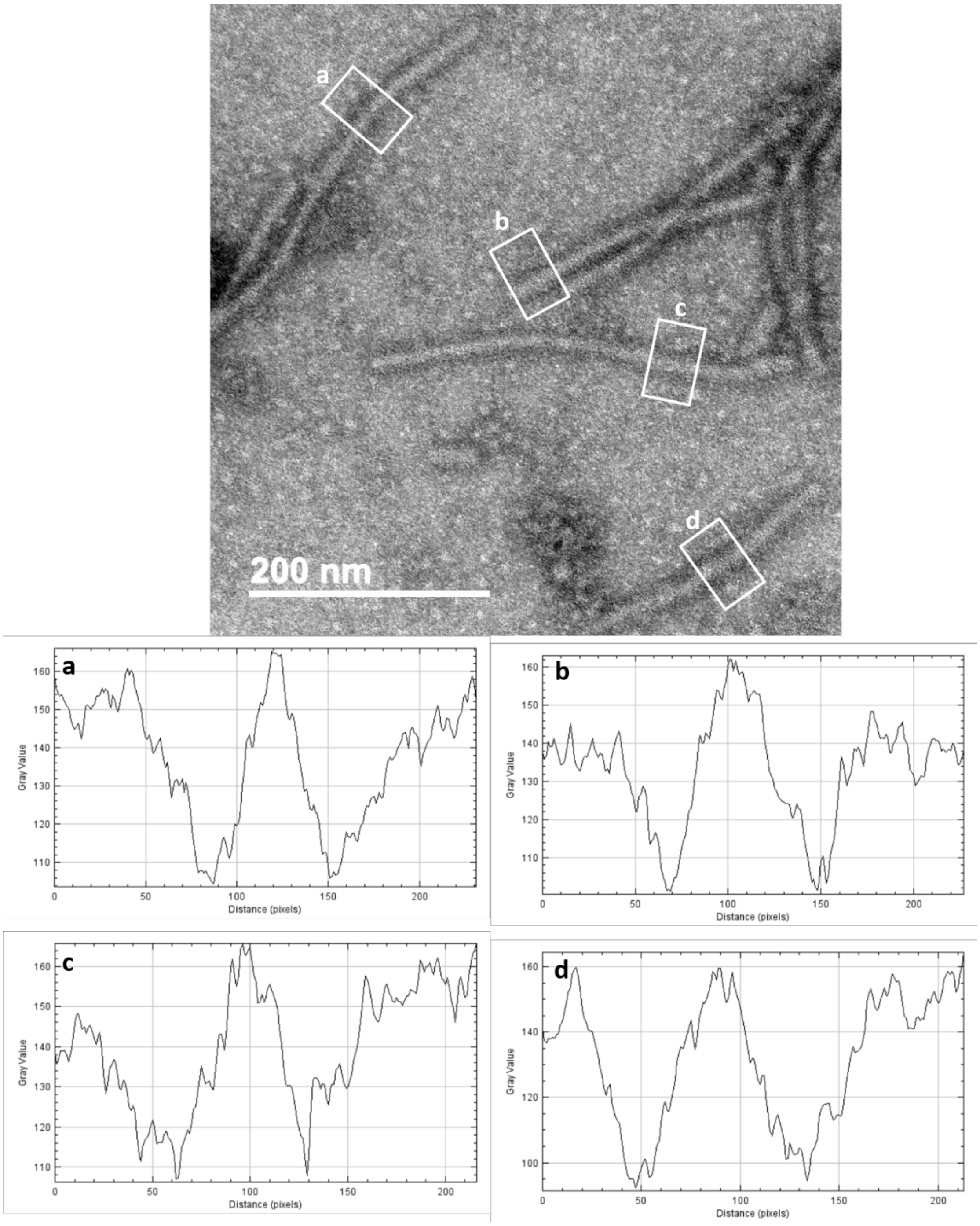


**Supplementary Figure 31:** Symmetrical assembly (blank). Top: TEM of (**4/8-PDMAc** + **8/4-PDMAc**) equimolar mixture containing $AgNO_3$ (negative staining with uranyl acetate). Bottom: grey scale analysis of the highlighted areas.

## 2.2. PATHWAY DEPENDENCY AND STABILITY OF JANUS NANORODS

### 2.2.1 Pathway dependency

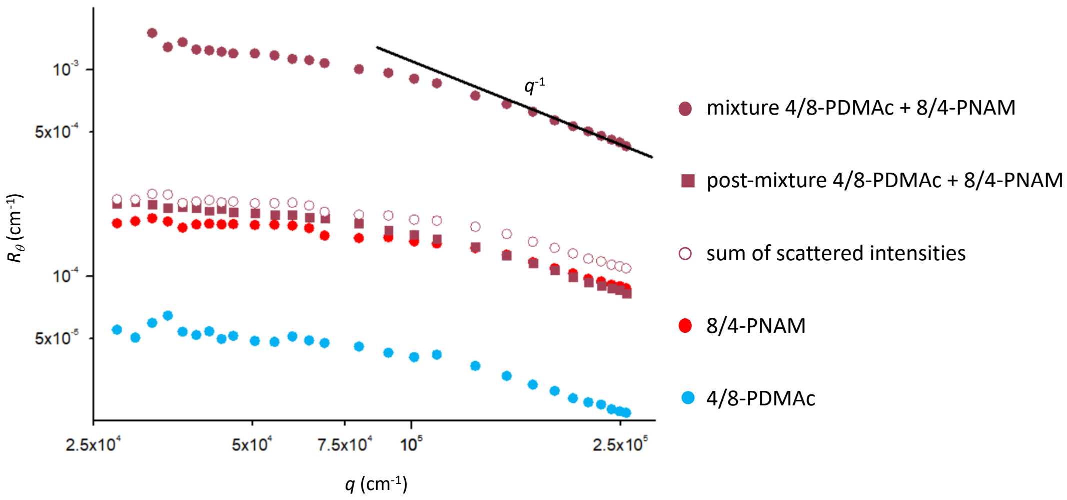


**Supplementary Figure 32:** Light scattering raw intensity for solutions of either **4/8-PDMAc**, **8/4-PNAM** or their equimolar mixtures: **4/8-PDMAc** + **8/4-PNAM**. The purple circles correspond to the classical preparation: water addition to the premixed polymers in DMSO (same data as Supplementary Figure 22). The purple squares correspond to post-mixing of the individually pre-assembled polymers (by water addition to **4/8-PDMAc** or **8/4-PNAM** in DMSO). The similar intensity for the post-mixture compared to the sum of intensities scattered by each individual polymer solution, indicates the absence of co-assembly, i.e. at least one of the pre-assembled polymers is kinetically frozen.

### 2.2.2 Time stability

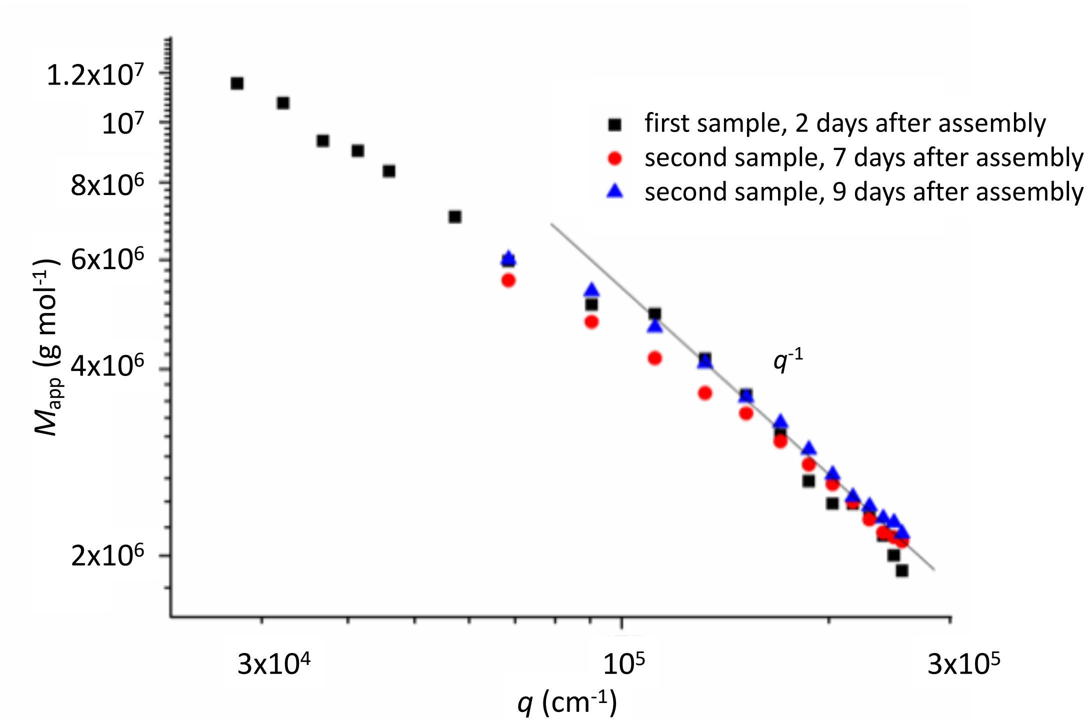


**Supplementary Figure 33:** Light scattering data: wave vector ($q$) dependency of the apparent molar mass of equimolar mixtures (**4/8-PDMAc** + **8/4-PNAM**) prepared by water addition to the premixed polymers in DMSO (1 g $L^{-1}$ in water/DMSO (99/1)). Duplicate experiments were performed and the measurements were performed at different times after water addition, showing reproducibility and absence of time evolution.

a)

b)

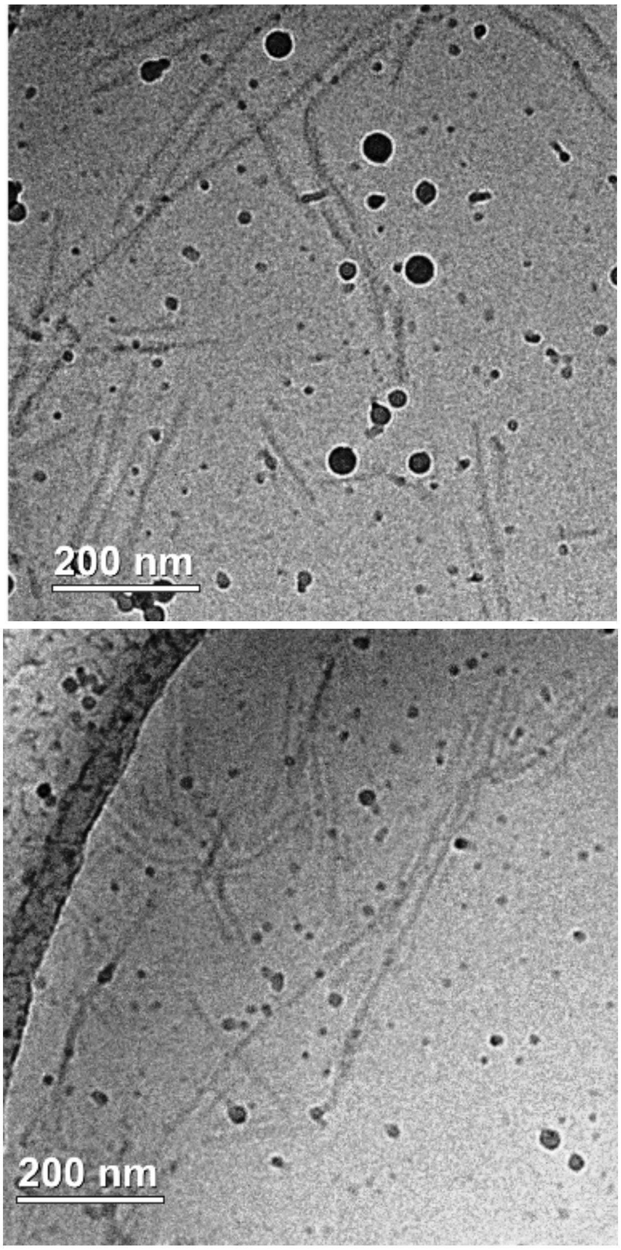


**Supplementary Figure 34:** Cryo-TEM images of Janus nanorods obtained from (**4/8-PDMAc** + **8/4-PNAM**) equimolar mixture, 12 hours (a) or 3 months (b) after preparation. 1 g $L^{-1}$ in water/DMSO (99/1). The dark spots are contaminations stemming from water crystals on the sample surface.

### 2.2.3 Temperature stability

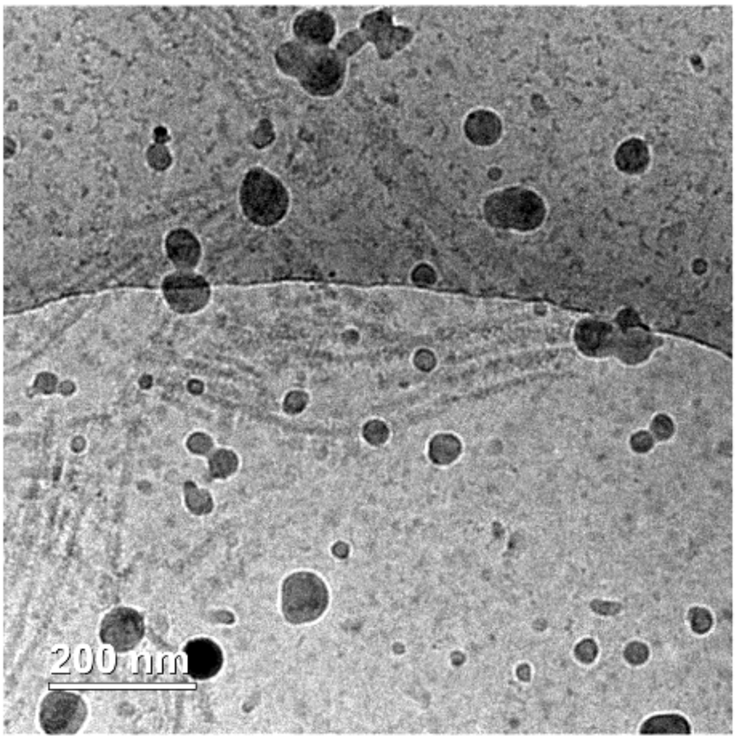


**Supplementary Figure 35:** Cryo-TEM images of Janus nanorods obtained from (**4/8-PDMAc** + **8/4-PNAM**) equimolar mixture, after heating at 80 °C for 1 hour. 1 g $L^{-1}$ in water/DMSO (99/1). Reference sample: Supplementary Figure 34. The dark spots are contaminations stemming from water crystals on the sample surface.

### 2.2.4 pH stability

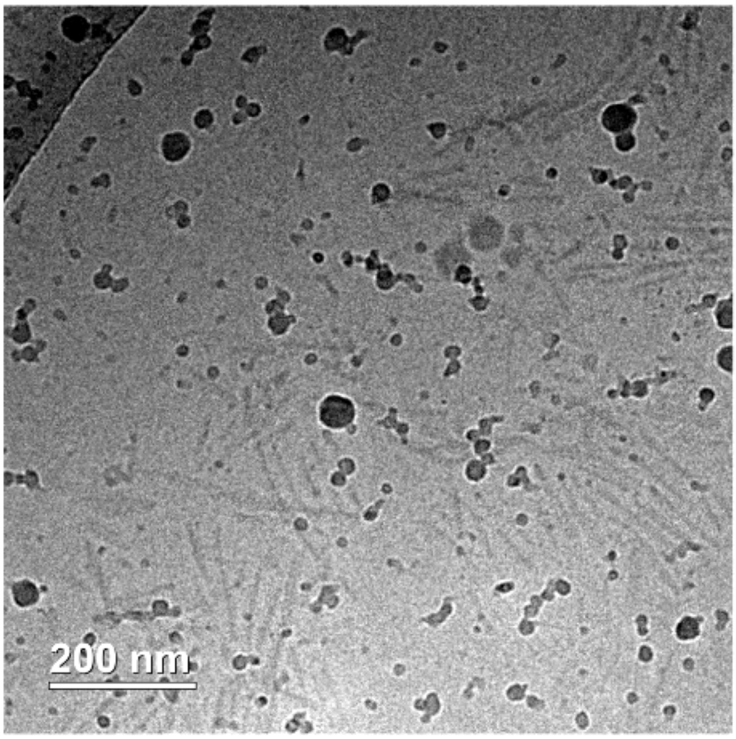


**Supplementary Figure 36:** Cryo-TEM images of Janus nanorods obtained from (**4/8-PDMAc** + **8/4-PNAM**) equimolar mixture, 4 hours after adjusting the pH to 10 with NaOH. 1 g $L^{-1}$ in water/DMSO (99/1). Reference sample: Supplementary Figure 34. The dark spots are contaminations stemming from water crystals on the sample surface.

## 2.3. CHARACTERIZATION OF THE EMULSIONS

### 2.3.1 Water/ethyl acetate emulsions: macroscopic observations

First, several solutions were prepared.

*Solution A*: PDMAc (synthesized from a non-functionalized RAFT agent, $M_{n,SEC}$ = 2500 g mol$^{-1}$, $Đ$ = 1.20) and PNAM (synthesized from a non-functionalized RAFT agent, $M_{n,SEC}$ = 3600 g mol$^{-1}$, $Đ$ = 1.15) were dissolved in water to prepare a 5 g L$^{-1}$ solution (molar ratio 1/1).

*Solution C*: a PDMAc-*b*-PNAM diblock copolymer (synthesized from a non-functionalized RAFT agent, $DP_{n(PDMAc)}$ = 35, $DP_{n(PNAM)}$ = 45, $M_{n,SEC}$ = 9500 g mol$^{-1}$, $Đ$ = 1.27) was dissolved in water to prepare a 5 g L$^{-1}$ solution.

*Solution D*: a **4/8-PDMAc** 100 g L$^{-1}$ DMSO solution and an **8/4-PNAM** 100 g L$^{-1}$ DMSO solution were mixed together in a 1/1 molar proportion (volume ratio of the solutions: 36/64). Pure water was added (0.5 mL h$^{-1}$) until a DMSO/water proportion of 1/99 (v/v). DMSO was removed by dialysis against water for 3 days using a dialysis membrane (MWCO = 1 kDa). The solution was concentrated to 5 g L$^{-1}$ by evaporation of water by a gentle air flow (room temperature and ambient pressure).

*Solution E*: Part of solution D (1 g L$^{-1}$) was freeze-dried and the powder was dissolved in water to prepare a 5 g L$^{-1}$ solution.

*Solution F*: a **4/8-PDMAc** 100 g L$^{-1}$ DMSO solution and an **8/4-PDMAc** 100 g L$^{-1}$ DMSO solution were mixed together in a 1/1 molar proportion (volume ratio of the solutions: 50/50). Pure water was added (0.5 mL h$^{-1}$) until a DMSO/water proportion of 1/99 (v/v). DMSO was removed by dialysis against water for 3 days using a dialysis membrane (MWCO = 1 kDa). The solution was concentrated to 5 g L$^{-1}$ by evaporation of water by a gentle air flow (room temperature and ambient pressure).

Then, emulsions were prepared from previous solutions. For all samples (A, C, D, E, and F): 1 µL, 5 µL, 10 µL, 50 µL, 100 µL, 200 µL or 400 µL of the 5 g L$^{-1}$ aqueous solutions described above were added into different tubes. Water was added to these tubes to a final volume of 1.6 mL. Finally, 0.4 mL of ethyl acetate was added to each tube to reach a constant 80/20 vol ratio of water/AcOEt. All the samples were shaken for 4 days on a shaking table and then visually observed at rest for up to 2 weeks.

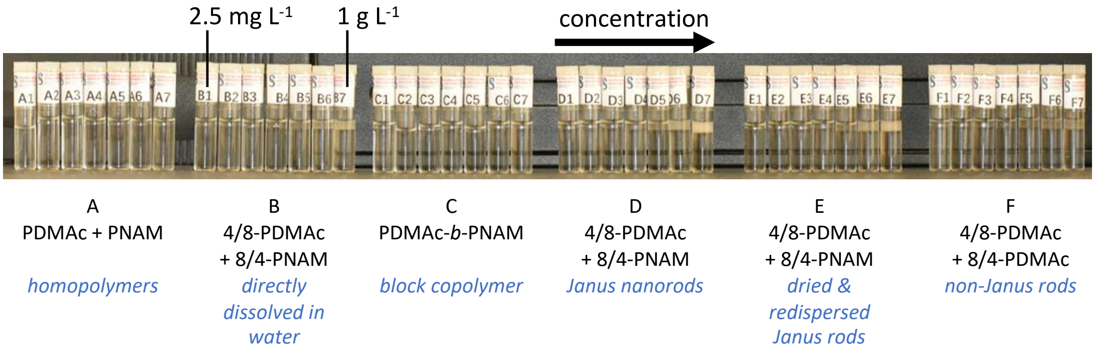


**Supplementary Figure 37:** Water/ethyl acetate (80/20) mixtures with various additives, 2 weeks after shaking. The numbers indicate concentration of the additive (1=2.5ppm; 2=12.5ppm; 3=25ppm; 4=125ppm; 5=250ppm; 6=500ppm; 7=1000ppm). The letters indicate the nature of the additive. The cloudy phase corresponds to ethyl acetate droplets that have creamed but have not coalesced (see Supplementary Figure 38).

### 2.3.2 Water/ethyl acetate emulsions: confocal imaging

1.6 mL of solutions C, D, E, F at 1.2 g $L^{-1}$ in water were prepared as described above. To each respective solution, 0.4 mL of ethyl acetate containing 10 ppm of Nile Red were added. The emulsions containing 1 g $L^{-1}$ of polymer were then formed by hand-shaking vigorously the tubes for 15 s and then vortexing them for 30 s at 2000 rpm. After creaming (which took 1-5 min in all cases), the emulsions were inserted between a concave slide and a cover slip that were hermetically sealed with a varnish. Confocal Laser Scanning Microscopy (CSLM) observations were performed at 25 °C with a Zeiss LSM800. The images of 9 tile assemblies (3x3) with a pixel size of 144 nm were obtained with a 25× water immersion objective (LD LCI Plan Apochromat) with a numerical aperture of 0.8 and a zoom of 0.5. An excitation wavelength of 561 nm and detection wavelengths range between 400 to 700 nm were used for the Nile Red detection.

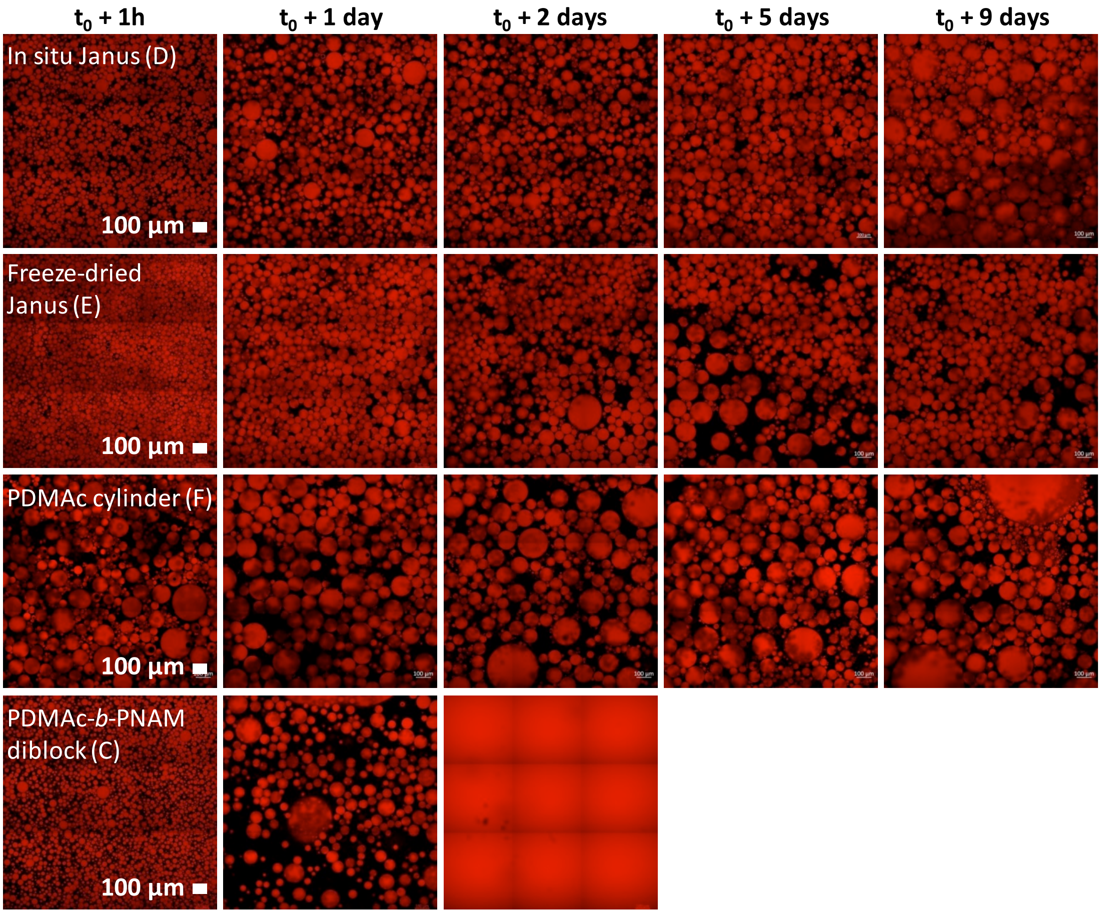


**Supplementary Figure 38:** Time-evolution of the emulsified part of water/ethyl acetate (80/20) mixtures with various additives observed by CSLM. Each line corresponds to a different additive (as indicated on the image at $t_0$ + 1 h). The ethyl acetate phase was marked with Nile Red. The droplets in red therefore correspond to ethyl acetate, whereas the water phase is black. The scale is the same for all images. For PDMAc-*b*-PNAM, macroscopic phase separation occurred after 2 days.

Supplementary Figure 38 reveals that the Janus nanorods, either prepared in situ (D) or freeze-dried and redispersed (E), lead to initially rather small and monodisperse ethyl acetate droplets which are well protected from coalescence even after 9 days. By comparison, homogeneous nanorods of PDMAc also hinder the coalescence of the ethyl acetate droplets, but do not afford the initial formation of small and rather monodisperse droplets. On the contrary, the PDMAc-*b*-PNAM diblocks initially favor the efficient dispersion of the ethyl acetate phase, but are hardly able to prevent coalescence. As a conclusion, only the Janus nanorods are able to form stable emulsions of finely dispersed ethyl acetate droplets because they combine the efficiency of cylinders to hinder coalescence (probably through a Pickering effect) and the ability of the diblock copolymers to initially disperse ethyl acetate with an energetically undemanding process (probably because of a reduction of the interfacial tension by their amphiphilic character).

### 2.3.3 Water/β-pinene emulsions: macroscopic observations

First, several solutions were prepared.

*Solution L*: PDMAc (synthesized from a non-functionalized RAFT agent, $M_{n,SEC}$ = 2500 g mol$^{-1}$, $Đ$ = 1.20) and P*n*BA (synthesized from a non-functionalized RAFT agent, $M_{n,SEC}$ = 4900 g mol$^{-1}$, $Đ$ = 1.17) were dissolved in dioxane to prepare a 5 g L$^{-1}$ solution (molar ratio 1/1).

*Solution M*: **4/8-P*n*BA** and **8/4-PDMAc** (molar ratio 1/1) were dissolved in dioxane to prepare a 5 g L$^{-1}$ solution.

*Solution N*: a P*n*BA-*b*-PDMAc diblock copolymer (synthesized from a non-functionalized RAFT agent, $DP_{n(PnBA)}$ = 31, $DP_{n(PDMAc)}$ = 35, $M_{n,SEC}$ = 2900 g mol$^{-1}$, $Đ$ = 1.89) was dissolved in dioxane to prepare a 5 g L$^{-1}$ solution.

*Solution O*: a **4/8-P*n*BA** 100 g L$^{-1}$ ethanol solution and an **8/4-PDMAc** 100 g L$^{-1}$ ethanol solution were mixed together in a 1/1 molar proportion (volume ratio of the solutions: 60/40). Pure dioxane was added at 50 °C (0.5 mL h$^{-1}$) until an ethanol/dioxane proportion of 1/99 (v/v). Solvents were removed by freeze-drying. The powder was re-dissolved in dioxane to prepare a 5 g L$^{-1}$ solution.

Then, emulsions were prepared from previous solutions. For all samples (L, M, N, and O): 10 µL of the 5 g L$^{-1}$ solutions were added into different tubes and then freeze-dried to remove dioxane. Water (1.6 mL) was added, and then 0.4 mL of β-pinene was added to each tube. All the samples were vortexed for 1 minute and then kept at rest for up to 2 weeks.

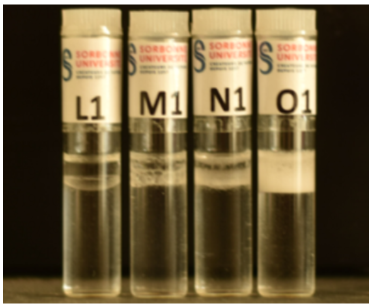


L : P*n*BA + PDMAc — *homopolymers*

M : 4/8-P*n*BA + 8/4-PDMAc — *directly dissolved in dioxane*

N : P*n*BA-b-PDMAc — *block copolymer*

O : 4/8-P*n*BA + 8/4-PDMAc — *Janus nanorods*

**Supplementary Figure 39:** Water/β-pinene (80/20) mixtures with various additives (25ppm), 2 weeks after vortexing. The letters indicate the nature of the additive. The cloudy phase corresponds to β-pinene droplets that have creamed but have not coalesced.

## 3. SUPPLEMENTARY REFERENCES

1. Qiu, X.-P., Tanaka, F. & Winnik, F. M. Temperature-induced phase transition of well-defined cyclic poly(*n*-isopropylacrylamide)s in aqueous solution. *Macromolecules* **40**, 7069–7071 (2007).
2. Han, S. *et al.* Oligo-urea with no alkylene unit self-assembles into rod-like objects in water. *Macromol. Rapid Commun.* 1800698 (2019).
3. Mellot, G. *et al.* Bisurea-functionalized RAFT agent: A straightforward and versatile tool toward the preparation of supramolecular cylindrical nanostructures in water. *Macromolecules* **51**, 10214-10222 (2018).
4. Perez-Folch, J., Subirana, J. A. & Aymami, J. Polar structure of *N,N'*-dimethylurea crystals. *J. Chem. Crystallogr.* **27**, 367-369 (1997).
